# The applied principles of EEG analysis methods in neuroscience and clinical neurology

**DOI:** 10.1186/s40779-023-00502-7

**Published:** 2023-12-19

**Authors:** Hao Zhang, Qing-Qi Zhou, He Chen, Xiao-Qing Hu, Wei-Guang Li, Yang Bai, Jun-Xia Han, Yao Wang, Zhen-Hu Liang, Dan Chen, Feng-Yu Cong, Jia-Qing Yan, Xiao-Li Li

**Affiliations:** 1 School of Systems Science, Beijing Normal University, Beijing, 100875 China; 2 College of Electrical and Control Engineering, North China University of Technology, Beijing, 100041 China; 3 School of Automation Science and Engineering, South China University of Technology, Guangzhou, 510641 China; 4 Department of Psychology, the State Key Laboratory of Brain and Cognitive Sciences, The University of Hong Kong, Hong Kong SAR, 999077 China; 5 HKU-Shenzhen Institute of Research and Innovation, Shenzhen, 518057 Guangdong China; 6 Department of Health Technology and Informatics, The Hong Kong Polytechnic University, Hong Kong SAR, 999077 China; 7 Department of Rehabilitation Medicine, the First Affiliated Hospital of Nanchang University, Nanchang, 330006 China; 8 Rehabilitation Medicine Clinical Research Center of Jiangxi Province, Nanchang, 330006 China; 9 Beijing Key Laboratory of Learning and Cognition, School of Psychology, Capital Normal University, Beijing, 100048 China; 10 School of Communication Science, Beijing Language and Culture University, Beijing, 100083 China; 11 Institute of Electrical Engineering, Yanshan University, Qinhuangdao, 066004 Hebei China; 12 School of Computer Science, Wuhan University, Wuhan, 430072 China; 13 School of Biomedical Engineering, Faculty of Electronic Information and Electrical Engineering, Dalian University of Technology, Dalian, 116081 Liaoning China; 14 Guangdong Artificial Intelligence and Digital Economy Laboratory (Guangzhou), Guangzhou, 510335 China

**Keywords:** Electroencephalogram analysis methods, Applied principles, Neuroscience, Diagnosis, Neurological diseases

## Abstract

Electroencephalography (EEG) is a non-invasive measurement method for brain activity. Due to its safety, high resolution, and hypersensitivity to dynamic changes in brain neural signals, EEG has aroused much interest in scientific research and medical fields. This article reviews the types of EEG signals, multiple EEG signal analysis methods, and the application of relevant methods in the neuroscience field and for diagnosing neurological diseases. First, three types of EEG signals, including time-invariant EEG, accurate event-related EEG, and random event-related EEG, are introduced. Second, five main directions for the methods of EEG analysis, including power spectrum analysis, time–frequency analysis, connectivity analysis, source localization methods, and machine learning methods, are described in the main section, along with different sub-methods and effect evaluations for solving the same problem. Finally, the application scenarios of different EEG analysis methods are emphasized, and the advantages and disadvantages of similar methods are distinguished. This article is expected to assist researchers in selecting suitable EEG analysis methods based on their research objectives, provide references for subsequent research, and summarize current issues and prospects for the future.

## Introduction

Neuroscience, also called brain science, is a discipline that explores the structure and function of the brain [1]. It is a typical inter-discipline that involves multiple disciplines, such as biology, psychology, information science, medicine, engineering, and artificial intelligence. Neuroscience has been developed for about 100 years and extensively applied to diagnose neurological disorders. With the development of research methods, the focus of neuroscience has gradually transitioned from brain structure to brain function in the past decade [2–4]. The brain nerve response is known as the core of cognitive production. Accurate identification of the brain nerve response can contribute to identifying important human cognitive functions, developing intelligent algorithms, and advancing medical developments regarding neurological diseases [3].

With the development of research tools for neuroscience, multiple neuroimaging tools are available for exploring brain function, including electroencephalography (EEG)/intracranial electroencephalography (iEEG), functional magnetic resonance imaging (fMRI), magnetoencephalography (MEG), positron emission tomography, and optogenetic techniques [4–7]. Among these methods, EEG/iEEG has been the most widely used tool for functional brain imaging due to its excellent temporal resolution and low equipment cost [8]. From the perspective of neurophysiology, EEG/iEEG reflects postsynaptic potential, which is generated when neurotransmitters bind to receptors on the postsynaptic membrane [9]. These postsynaptic potentials generate electric fields around neurons. Once sufficient neurons are activated, electroneurographic signals with specific patterns can be captured through a voltage amplifier. Owing to the shorter spatial distance between iEEG and neuronal groups, iEEG has higher accuracy and signal-to-noise ratio compared to EEG [9]. The electric signals captured by EEG have poor spatial resolution and signal-to-noise ratio since they are passed through the skull. However, EEG is a non-invasive technology, so it can be applied in a wider range of scenarios [8]. The information captured by both methods is the discharge of neuronal groups, so the capture equipment in both cases is a voltage amplifier, and the captured signals have basically the same manifestation. Hitherto, EEG/iEEG has been extensively applied to research diverse aspects of brain function, including attention [10], memory [11], language [12], emotions [13], and brain function disorders [14].

Although EEG/iEEG has good practicality, its application requires a certain foundation in signal processing technologies due to the complex representation of EEG signals; this leads to a problem in that some researchers lack clarity in selecting and applying analytical methods for EEG/iEEG. Therefore, we attempted to provide a brief introduction to commonly used EEG signal processing methods in this article. In this review, we first comprehensively classified EEG signals based on their characteristics. Next, we introduced commonly used analytical methods for EEG in terms of characteristics such as power spectrum and connectivity, and presented their adaptability to various types of EEG to assist researchers in method selection. We also summarized current issues and prospects for the future, which is expected to expedite the application of EEG/iEEG in brain science and neurological disease research.

## Types of EEG signals

Generally, in research articles, especially those on neurological disease, EEG is classified based on the research subjects. For instance, in sleep study, EEG is classified into EEG during wakefulness and sleep EEG [15]; in epilepsy-related study, EEG is sub-divided into interictal EEG, preictal EEG, ictal EEG, and postictal EEG [16]; and in research on event-related potentials, EEG is categorized into resting-state EEG and task-state EEG [17]. In addition, it can be classified according to the shape of the EEG itself. For example, it can be divided into delta, theta, and alpha based on frequency [18, 19] or slow wave, fast wave, sharp wave, and spike wave based on shape.

**Fig. 1:**
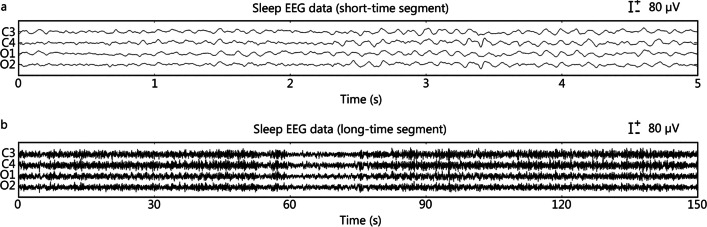
Example of time-invariant electroencephalogram (EEG) based on sleep EEG. The data come from the C3, C4, O1, and O2 channels (10–20 system). **a** EEG data for a short sleep time of 5 s. **b** EEG data for a long sleep time of 150 s

**Fig. 2:**
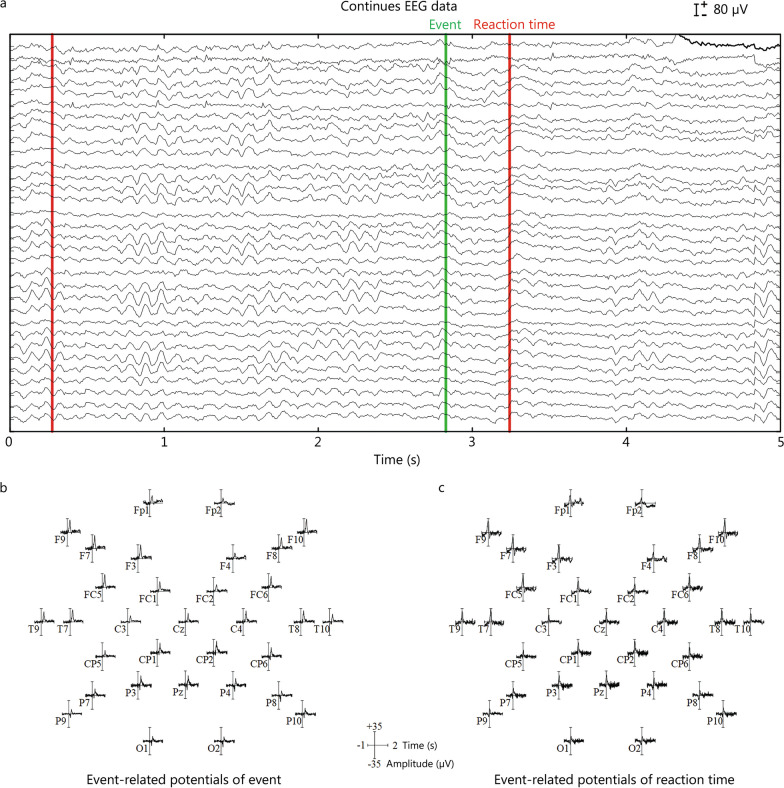
Example of time-invariant electroencephalogram (EEG) based on event-related potentials. **a** A 5-second period of continuous EEG data with an event marker. **b** Corresponding event-related potentials of all channels obtained by superimposing EEG signals with all “Event” markers taken as the zero time. **c** Corresponding event-related potentials of all channels obtained by superimposing EEG signals with all “Reaction time” markers taken as the zero time

**Fig. 3:**
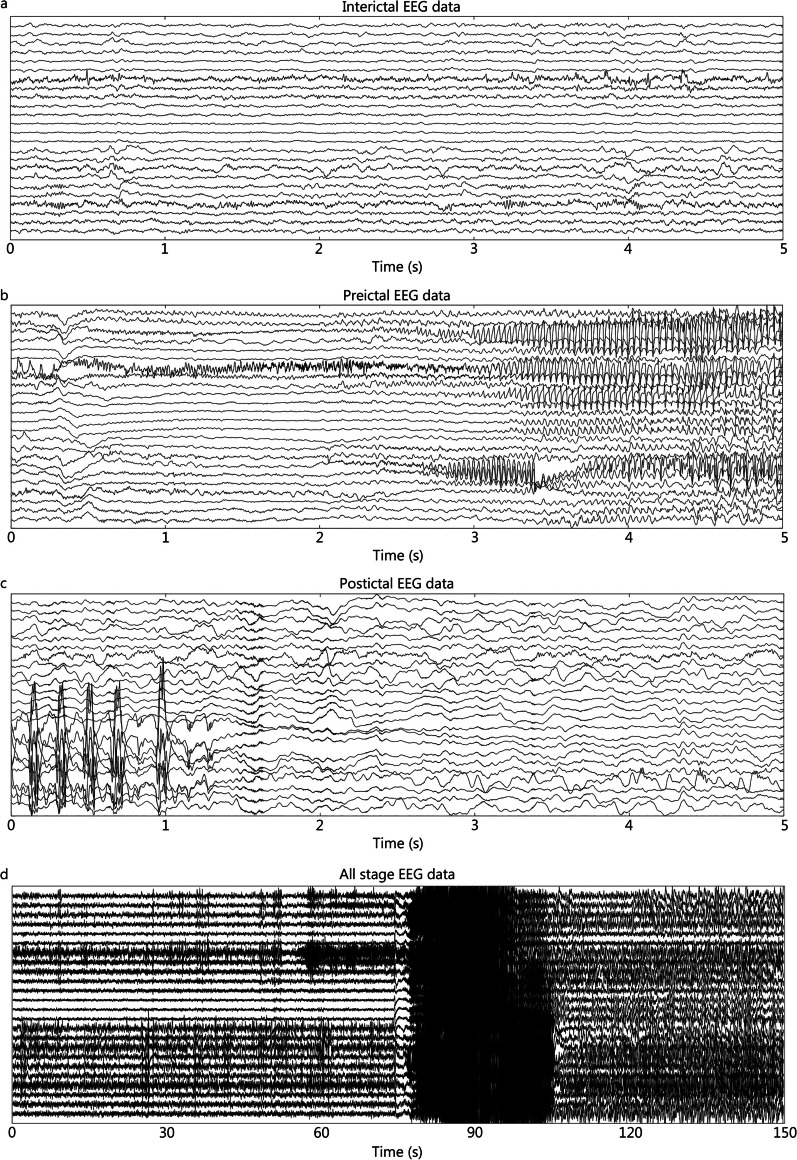
Example of time-invariant electroencephalogram (EEG) based on epilepsy EEG data. **a** A 5-second period of EEG data between epileptic seizures. **b** A 5-second period of EEG data in the early stage of an epileptic seizure. **c** A 5-second period of EEG data in the late stages of an epileptic seizure. **d** A 150-second period of EEG data from all stages of an epileptic seizure

However, from the perspective of EEG analysis, we believe that EEG can be classified into the following three categories. (1) An EEG in which the functional state of the brain remains unchanged over time is called a time-invariant EEG for short [20]. In this type of EEG, the state of the brain does not show significant changes during the capture process, for example, a resting-state EEG without psychological activity [16]. Alternatively, some changes in brain characteristics are not included among the main features to be studied. For example, in epilepsy research, researchers pay more attention to the pathological EEG; in this case, the interictal period without epileptic discharge can also be considered a time-invariant EEG [16]. Figure 1 is an example of sleep EEG. During sleep, the EEG is in a stable state for a long time. Figure 1a shows an EEG of a 5-s period of sleep, while Fig. 1b shows a 150-s sleep EEG. Although the EEG is unstable, relatively stable data segments can be found within the unstable EEG by analyzing these two segments. (2) Accurate event-related EEG can be regarded as an extension of event-related potentials; it refers to the EEG induced by a certain event where the induction time of the event is definite. EEG with time-varying characteristics caused by stimuli with a definite time, such as visuoauditory, transcranial magnetic stimulation, and electrical cortical stimulation [17]. Figure 2 is an example of event-related potential. Events will appear at a clear time, so accurate brain electrical responses can be obtained through time. Figure 2a is a section of an EEG containing event-related potentials, which marks the exact moment when the event occurred. Figure 2b shows the EEG response after superimposing multiple event-related potentials with the event as the 0 moment. Figure 2c shows the EEG response after the superposition of multiple event-related potentials, with the reaction time as the 0 moment. (3) Random event-related EEG refers to an EEG induced by a certain event in which the induction time of the event is random and cannot be determined. In research on diseases such as epilepsy or Parkinson’s disease, pathological EEG is triggered by abnormal neural activity in the lesion area, but the timing of pathological induction is difficult to determine, resulting in a time-varying EEG [19]. Figure 3 displays the EEG signals of an epilepsy patient. Figure 3a shows the EEG signal during the interictal period, while Fig. 3b, c show the EEG signal in the early and late stages of the seizure, respectively. Figure 3d shows the EEG signal of the entire seizure process. Epilepsy is a random event, so the time of occurrence of the event needs to be retrospectively located after event onset, which poses challenges to the real-time analysis of epilepsy EEG data. However, Fig. 3a, c show that epileptic EEG is still relatively stable within a period. In Fig. 3d, the data observed during the interictal and postictal periods, which represent two stable stages, reveals a significant difference.

It should also be noted that the main classification criteria for these three types of EEG were based on the EEG features to be analyzed, and specific analysis is required according to the features of interest. In sleep disorder research, we can consider stage N1 EEG as a time-invariant EEG. However, if the study target is a dream or memory, the main characteristics to be studied may also change during the N1 phase, and this EEG should be classified as a random event-related EEG. Therefore, researchers should accurately identify the target features to be studied before selecting an analytical method.

## Common EEG analysis methods

In this section, the extraction methods of common brain functional features based on the characteristics of EEG signals are introduced.

### Power spectrum analyses

The power spectrum is a very common analytical method in EEG analysis that can analyze the energy changes of various frequency components in EEG signals. This method can be applied in studies on brain science and neurological diseases that can trigger EEG energy changes upon state changes, such as sleep stage changes, seizures, and emotional changes. Multiple power analysis methods are available for selection, such as the fast Fourier transform (FFT), Welch, and autoregressive (AR) model, with different characteristics and usage limitations. The articles in Table 1 covered a range of topics related to EEG analysis, including sleep onset, transitions between sleep stages, classification of neurological disorders, detection of post-stroke EEG signals, analysis of EEG background activity in autism and dyslexia, and the impact of various factors such as focused ultrasound stimulation, cognitive impairment in diabetes, and neurofeedback training in autism [20–41].

**Table 1:** Applications of power spectrum analyses

References	Data type	Subjects	Method	Disease/state	Application	Effect evaluation
Ogilvie et al. [20]	EEG	18–25 years (male = 1, females = 8)	FFT	Sleep stages	Used the FFT method to reflect the energy changes during the onset and phase transition of sleep	During the transition from stage 2 to REM sleep, observable systematic changes in EEG power density were reported across four standard frequency bands
Hadjiyannakis et al. [21]	EEG	Normal (*n* = 9)	FFT	Sleep stages	Conducted an in-depth study of sleep 20 years ago using the FFT method, which reflected energy changes during the onset and staging transitions of sleep	Sleep onset was identified in this study by the cessation of responses coupled with sharp increases in EEG synchronization
Sun et al. [22]	EEG	SZ (males = 36, females = 18)	FFT	SZ	Adopted the FFT method to explore the EEG features of schizophrenic patients	An average accuracy of 96.34% was attained using FFT in this study
Behnam et al. [23]	EEG	Autism disorders (*n* = 10)	FFT	Autism disorders	Utilized the FFT method to analyze EEG differences in autistic patients with different electrodes and achieved significant performance at the FP1, F3 position	STFT-BW demonstrated an 82.4% discrimination rate between normal and autistic subjects using the Mahalanobis distance, whereas FFT and STFT did not yield significant results
Djamal et al. [24]	EEG	Stroke (*n* = 25) Normal (*n* = 25)	FFT	Stroke	Improved the recognition accuracy of a one-dimensional (1D) CNN for the EEG signals of stroke patients	Utilizing FFT for identification was suggested to enhance accuracy by 45–80% compared to relying solely on 1D CNN, according to the findings in this article
Farihah et al. [25]	EEG	Dyslexic (boys = 4)	FFT	Dyslexic	Applied the FFT method to explore the EEG characteristics of dyslexic patients	Distinct differences in hemispheric activation during the construction of sentences and nonsense sentences were observed between poor and capable dyslexic subjects, as reported in this study
Melinda et al. [26]	EEG	From King Abdul Aziz University (KUA)	FFT	ASD	Analyzed EEG differences in epileptic patients through the FFT approach	The article highlighted alterations in the PSD values, noting an increase in the alpha and beta sub-bands for normal EEG signals and a decrease for autistic EEG signals
Bian et al. [27]	EEG	MCI (*n* = 16) Normal (*n* = 12)	Welch	Amnestic MCI in diabetes	Achieved early identification of mild cognitive dysfunction using Welch’s method	Proposed indices derived from resting-state EEG recordings were proposed to serve as tools for monitoring cognitive function in diabetic patients and aiding in diagnosis, according to the claims in this article
Yuan et al. [28]	EEG	Normal rats (males = 5)	Welch	Transcranial ultrasound stimulation	Studied the changes in the brain function of animals responding to different intensities of transcranial ultrasound stimulation through Welch’s method	The article suggested that power spectrum analysis holds significant reference value for brain stimulation, providing estimates of the extent of stimulation or inhibition of excitement
Wang et al. [29]	EEG	ASD (males = 14, females = 4)	Welch	ASD	Investigated the impacts of neurofeedback training on the cognitive function of autistic children based on the changes in frequency band energy	Neuro-feedback was presented as an effective method for altering EEG characteristics associated with ASD in this article
Göker [30]	EEG	Migraine (males = 5, females = 13) Normal (males = 9, females = 12)	Welch	Migraine	Adopted the Welch method to improve the accuracy of automatic migraine detection	The highest performance, with a 95.99% accuracy, was reported for the BiLSTM deep learning algorithm using 128 channels in this article
Hu et al. [31]	EEG	Normal (*n* = 3)	Welch	Hypoxia	Employed Welch’s method in combination with BP and SVM classifiers for hypoxic EEG classification, achieving an accuracy of 94.2% (BP classifier) and 92.5% (SVM classifier)	Distinguishing hypoxic EEG from normal EEG in individuals was demonstrated in this study
Wijaya et al. [32]	EEG	Stroke (*n* = 10)	Welch	Stroke	Used the Welch method for the classification of patients with acute ischemic stroke, similar to CT scan results	All BSI calculations exceeded those of healthy subjects (0.042 ± 0.005), indicating acute ischemic stroke in all subjects, as presented in this article
Cornelissen et al. [33]	EEG	Infants (*n* = 36)	Multitaper	General anesthesia in infants	Applied this method to study the effects of drugs under anesthesia on neonatal brain function	The article emphasized the necessity of age-adjusted analytical approaches for developing neurophysiology-based strategies in pediatric anesthetic state monitoring
Yang et al. [34]	EEG	Normal (*n* = 35)	Multitaper	SSVEP	Improved the accuracy of 40-class SSVEP using the multitaper method	The proposed method was asserted to effectively enhance the performance of a training-free SSVEP-based BCI system and balance recognition accuracy across different stimulation frequencies
Oliva et al. [35]	EEG	Bonn dataset	Multitaper	Epilepsy	Adopted the multitaper method for epilepsy detection with the assistance of different classifiers	This article reported achieving the highest accuracy for both binary (100%) and multiclass (98%) classification problems
Oliveira et al. [36]	EEG	DREAMs dataset	Multitaper	Sleep	Used the multitaper method for the automatic detection of KC waveforms in a sleep EEG and obtained favorable results	The method for automatic KC detection was asserted to improve detection metrics, particularly F1 and F2 scores, according to the claims in this article
Mohammadi et al. [37]	EEG	Normal (males = 6, females = 4)	AR	Person Identification	Achieved personal identification using the AR model	Classification scores ranging from 80 to 100% were achieved, revealing the potential of the approach for personal classification/identification
Perumalsamy et al. [38]	EEG	Normal (*n* = 5)	AR	Sleep spindles detection	Extracted sleep feature waves through the AR model	The algorithm's effectiveness in detecting sleep spindles and revealing alpha and beta band activities in EEG was demonstrated in this article
Saidatul et al. [39]	EEG	Normal (males = 5)	AR	Relaxation and mental stress condition	Applied AR modeling techniques to analyze EEG differences between relaxed and stressed states	A maximum classification accuracy of 91.17% was reported in this study
Lawhern et al. [40]	EEG	Normal (*n* = 7)	AR	Artifacts detection	Adopted the AR model to remove artifacts in EEG signals	AR modeling was suggested as a useful tool for discriminating artifact signals within and across individuals, according to the claims in this article
Mousavi et al. [41]	EEG	Bonn dataset	AR	Epilepsy	Used the AR model to automatically detect epileptic events in EEG signals	Correct classification scores in the range of 91% to 96% for epilepsy detection were reported in this study

*AR* autoregressive, *ASD* autism spectrum disorder, *BCI* brain-computer interface, *BiLSTM* bidirectional long short-term memory, *BP* backpropagation, *BSI* brain symmetry index, *CNN* convolutional neural network, *CT* computed tomography, *DREAM* dialogue-based reading comprehension examination, *EEG* electroencephalography, *FFT* fast Fourier transform, *KC* k-complex, *MCI* mild cognitive impairment, *PSD* power spectral density, *REM* rapid eye movement, *SSVEP* steady state visually evoked potential, *SVM* support vector machine, *SZ* schizophrenia, *STFT-BW* short time Fourier transform at bandwidth of total spectrum

#### FFT

FFT is a fast algorithm for computing discrete Fourier transform (DFT) [42]. DFT:

1 $$X\left(k\right)=\sum_{n=0}^{N-1}x\left(n\right){e}^{-i2\pi nk/N},\mathrm{k}=0,\dots ,\mathrm{N}-1$$

Here, $X\left(k\right)$ denotes the DFT, $N$ represents the length of the available data, $x\left(n\right)$ refers to the input signal in the time domain, $e$ signifies the exponential operation, $i$ denotes the imaginary part, and $k$ represents the sampling frequency. The calculation process of Equation (1) is known as the FFT algorithm. Using the symmetric and periodic nature of the exponential factor in the DFT calculation equation, FFT can reduce repetitive calculations [42]. FFT calculations have a high-frequency resolution but are also easily affected by EEG signal noise, so an average period method has been proposed for improvement.

The average period method splits the original signal into *N* non-overlapping consecutive segments and then calculates the periodogram of each segment individually, all of which are finally averaged [43]. Through the window technique, averaging *N* periodograms can reduce the variance of the power spectral density estimation, but spectral leakage easily occurs because signal segmentation leads to increased boundaries of the data, while Fourier transform has a poor ability to process these data boundaries [44].

#### Welch

The Welch method has two improvements that enhance resolution and reduce errors in results compared to the average period method. First, this method allows overlap between data segments. Second, the Hamming window function is used for each segment instead of the rectangular window function, which ameliorates the potential distortion caused by too many rectangular windows [45]. ${\{x}_{l}\left(n\right)\},l=1,\dots ,S$ refer to data segments, and $M$ represents the length of each segment. The overlapping coefficient is usually set as 50% ($M$/2). The Welch spectrum estimate is given by the following equation:

2 $${\widehat{P}}_{l}\left(f\right)=\frac{1}{M}\frac{1}{P}{\left|\sum_{n=1}^{M}v\left(n\right){x}_{l}\left(n\right){e}^{-i2\pi nf}\right|}^{2}$$

3 $${\widehat{P}}_{w}\left(f\right)=\frac{1}{S}\sum_{l=1}^{S}{\widehat{P}}_{l}\left(f\right)$$

Here, ${\widehat{P}}_{l}\left(f\right)$ represents the periodogram estimate of segment $l$ and $v\left(n\right)$ denotes the window function. $P$ refers to the general average of $\left|{v}_{\left(n\right)}^{2}\right|$: $P=1/M{\Sigma }_{n=1}^{M}{\left|{v}_{\left(n\right)}^{2}\right|}^{2}$, $e$ signifies the exponential operation, $i$ denotes the imaginary part, $f$ represents the sampling frequency, ${\widehat{P}}_{w}\left(f\right)$ refers to the Welch power spectral density estimate, and $S$ signifies the number of segments.

Currently, Welch’s method is one of the most widely used power spectrum analysis methods because it reduces the influence of boundary effects on the power spectrum, providing more stable power spectral results than FFT/short-time Fourier transform (STFT) methods.

#### Multitaper

Multitaper can solve the bias and variance problems of nonparametric spectral estimation simultaneously in an optimal manner [46]. Windowing the signal using different tapers allows multiple independent estimates to be derived from the same signal since the different windows are uncorrelated with each other.

Assuming that ${X}_{k}={\{{x}_{1,k},{x}_{2,k},\dots ,{x}_{p,k}\}}^{T}$ is the signal sequence, where *p* denotes the number of channels and *k* signifies the length of the sequence, the multitaper of the channel $l,m$ is calculated as follows:

4 $${\widehat{S}}_{l,m}^{mt}\left(f\right)=\frac{1}{K}\sum_{i=1}^{K}{\widehat{S}}_{l,m}^{\left(i\right)}\left(f\right)$$

5 $${\widehat{S}}_{l,m}^{\left(i\right)}\left(f\right)=\Delta \left[\sum_{k=0}^{N-1}{h}_{k}^{\left(i\right)}{x}_{l,k}{e}^{-i2\pi kf\Delta }\right]\left[\sum_{k=0}^{N-1}{h}_{k}^{\left(i\right)}{x}_{m,k}{e}^{-i2\pi kf\Delta }\right]$$

Here, *K* refers to the cross-spectrum estimate, *N* represents the sequence length, ${\widehat{S}}_{l,m}^{\left(i\right)}\left(f\right)$ signifies the $k$th direct cross-spectrum estimate of the channel $l,m$, $\Delta$ indicates the sampling interval, $f$ represents the sampling frequency, $e$ signifies the exponential operation, $i$ denotes the imaginary part, and ${h}_{k}^{\left(i\right)}$ represents taper. Since the final result is obtained by processing multiple tapers, the problem of information loss caused by single-scale analysis can be reduced.

The multitaper method is a modified Welch’s method that provides features similar to those of the STFT and Welch’s methods, but its stability is improved and the number of parameters to be determined is reduced because it uses multiple tapers for superposition. For instance, in the well-known tool Fieldtrip, multitaper is employed as the major power spectrum analysis method [47].

#### AR model

The AR model can achieve the linear prediction modeling of the original signal as with the signal $x\left(n\right),0\le n\le (N-1)$ deemed as white noise with a mean value of 0 and a variance equal to ${\sigma }^{2}$. The signal’s amplitude during a specific period is determined by aggregating the various amplitudes from preceding signals and incorporating the estimation error. The model’s order, or filter, is contingent upon the quantity of AR coefficients employed.

6 $$x\left(n\right)=-\sum_{k=1}^{p}a\left(k\right)x\left(n-k\right)+w\left(n\right)$$

Here, $a\left(k\right)$ represents the coefficients of the AR model, $w\left(n\right)$ signifies white noise with a variance equal to ${\sigma }^{2}$, and $p$ refers to the model order. The AR(P) model can be characterized in terms of parameters $\left\{a\left[1\right],a\left[2\right],\dots ,a\left[p\right],{\sigma }^{2}\right\}$. Power spectral density:

7 $${P}_{AR}\left(f\right)=\frac{{\sigma }^{2}}{{\left|A\left(f\right)\right|}^{2}}$$

$A\left(f\right)=1+{a}_{1}\mathrm{exp}\left(-j2\pi f\right)+{\dots +a}_{p}\mathrm{exp}\left(-j2\pi fp\right).$AR model parameters can be derived using Burg or least squares [48]. The AR model can process the short-term signal in contrast to the FFT. The AR model is less used because of its high coefficient requirement, but its scalability is so excellent that it has been used in many researches [37–41].

### Time–frequency analyses

Since the EEG of human beings in a task state shows time-dependent changes, a time–frequency analysis is quite suitable for EEG analysis. For short-term signals, time–frequency analysis can replace power spectrum analysis to characterize the signals in two dimensions. The commonly used methods for time–frequency analysis include STFT, the wavelet transform (WT), empirical mode decomposition (EMD), and the Wigner-Ville distribution (WVD). The articles in Table 2 investigated various methods for EEG signal analysis, including the use of rational discrete STFT and deep learning for epileptic seizure classification, a hybrid approach for alcohol and control EEG signal classification, connectivity analysis in autism disorders using STFT and coherence values, drowsiness detection based on relative band power and STFT, automatic sleep stage classification using time–frequency images, and detection of deception using smoothed pseudo WVD, among other techniques [49–68].

**Table 2:** Applications of time–frequency analyses

References	Data type	Subjects	Method	Disease/state	Application	Effect evaluation
Samiee et al. [49]	EEG	Bonn dataset	STFT	Epilepsy	Utilized the STFT method to extract EEG data during seizure periods and non-seizure periods	The proposed method in this study was asserted to surpass competing techniques in classification accuracy while offering a compact representation of EEG time series
Beeraka et al. [50]	EEG	Bonn dataset	STFT	Epilepsy	Detected epileptic seizures in patients using the STFT method	Using CNN and BiLSTM models, this article reported an average classification accuracy of 93.9% and 97.2%, respectively
Bajaj et al. [51]	EEG	Normal (*n* = 120)	STFT	Alcohol	Employed the STFT algorithm to classify EEG features in patients with alcoholic encephalopathy	Experimental outcomes and comparisons with state-of-the-art algorithms led the article to claim the superior performance of the proposed method over competing algorithms
Sheikhani et al. [52]	EEG	Autism disorder (males = 9, female = 1)	STFT	Autism disorder	Adopted the STFT algorithm to analyze the brain activity of autistic patients	The beta band (14–34 Hz) was highlighted in this article for demonstrating an 82.4% discrimination rate between the two groups
Krishnan et al. [53]	EEG	DROZY database	STFT	Drowsiness	Utilized the STFT algorithm to detect and categorize two sleep states, “drowsy” and “alert”	Employing the KNN classifier, the article achieved classification accuracies of 96.1% (dataset 1) and 95.5% (dataset 2)
Bajaj et al. [54]	EEG	Normal (*n* = 8)	WVD	Sleep stages	Applied the WVD to extract the characteristics of sleep stages	Effectiveness in classifying sleep stages from EEG signals was demonstrated in this article through the proposed method
Yan et al. [55]	iEEG	Epilepsy (*n* = 21)	WVD	Epilepsy	Detected seizures in patients using the WVD	This article showcased a satisfactory sensitivity of 94.26%, a specificity of 96.34%, and a very short delay time of 0.56 s
Ebrahimzadeh et al. [56]	EEG	Normal (*n* = 32)	WVD	Deception	Developed a P300-based deception detection method using the WVD	The method presented in this article claimed to detect deception with an accuracy of 89.73%, surpassing the performance of previously reported methods
Khare et al. [57]	EEG	SZ (*n* = 49) Normal (*n* = 32)	WVD	SZ	Used the WVD approach to automatically detect EEG signals in schizophrenic patients	An accuracy of 93.36% was achieved in this article using the smoothed pseudo-WVD-based time–frequency representation and CNN model
Faust et al. [58]	EEG	Bonn dataset	WT	Epilepsy	Adopted the WT algorithm to develop an assisted seizure detection system	The article asserted that the method presented is the most effective for the automated EEG-based diagnosis of epilepsy
Gandhi et al. [59]	EEG	Epilepsy Normal	WT	Epilepsy	Developed an expert model for epileptic activity detection using the WT method	Detection accuracy of 99.33%, along with a sensitivity of 99.6% and specificity of 99%, was claimed in this article
Anuragi et al. [60]	EEG	Alcoholic and non-alcoholic (*n* = 122)	WT	Alcohol	Applied the WT algorithm for the automatic detection of alcoholism	The LS-SVM using a polynomial kernel was reported as the best performer in this article, achieving an accuracy of 99.17%, sensitivity of 99.17%, and specificity of 99.44% with a tenfold cross-validation technique
Murugappan et al. [61]	EEG	Normal (*n* = 20)	WT	Emotion	Reported the application of the WT algorithm for emotional recognition	Maximum average classification rates of 83.26% using KNN and 75.21% using LDA were achieved in this article, outperforming conventional features
Adeli et al. [62]	EEG	Epilepsy (*n* = 2)	WT	Epilepsy	Analyzed the EEG signals of epileptic patients with the WT algorithm	Wavelet analyses of EEGs from a patient population were suggested in this article to potentially indicate physiological processes during epilepsy onset
Hamad et al. [63]	EEG	Bonn dataset	WT	Epilepsy	Utilized the WT algorithm to extract multiple epileptic features	Extracting 10 features from EEG signals based on discrete WT, this article aimed to improve accuracy in classifying EEG signals for epilepsy detection
Li et al. [64]	EEG	Bonn dataset Epilepsy (*n* = 6)	EMD	Epilepsy	Analyzed automated seizure detection using the EMD algorithm	This article reported sensitivities of 97.00% and 98.00%, and specificities of 96.25% and 99.40% for interictal and ictal EEGs and normal and ictal EEGs, respectively, on Bonn datasets
Siuly et al. [65]	EEG	SZ (*n* = 49) Normal (*n* = 32)	EMD	SZ	Applied the EMD algorithm for the automated detection of SZ	The ensemble bagged tree was identified as the best classifier in this article, achieving a 93.21% correct classification rate for SZ
Chen et al. [66]	EEG	Awareness (samples = 558) Anesthesia (samples = 558)	EMD	Anesthesia	Adopted the EMD algorithm to classify anesthetized and awake states	The article claimed that the IMF performed the best, with an AUC of 0.993 for FFT (or 0.989 for Hilbert transform)
Babiker et al. [67]	EEG	Normal (males = 38, females = 5)	EMD	Situational interest	Detected the situational interest of students in the learning process with the application of the EMD algorithm	While SVM achieved high accuracies of 93.3% and 87.5% for two datasets using features from four EEG channels, the KNN classifier achieved comparable accuracies of 87.5% and 86.7% for the same datasets using a single EEG channel, as reported in this article
Priya et al. [68]	EEG	Alcoholic and non-alcoholic (*n* = 122)	EMD	Alcohol	Employed an EMD algorithm to differentiate between alcoholic EEG and normal EEG	Accuracy results for an LS-SVM classifier with polynomial and RBF kernels were stated as 96.67% and 97.92%, respectively, in this article

*AUC* area under the curve, *BiLSTM* bidirectional long short-term memory, *CNN* convolutional neural network, *EEG* electroencephalography, *EMD* empirical mode decomposition, *FFT* fast Fourier transform, *iEEG* intracranial electroencephalography, *IMF* intrinsic mode function, *KNN* k-Nearest Neighbors, *LDA* linear discriminant analysis, *LS-SVM* least-squares support vector machine, *RBF* radial basis function, *STFT* short-time Fourier transform, *SVM* support vector machine, *SZ* schizophrenia, *WT* wavelet transform, *WVD* Wigner-Ville distribution

#### STFT

The STFT is a technique that divides long-term signals into shorter segments of uniform length. It then computes the Fourier transform separately for each of these shorter segments. The Fourier transform is defined as follows:

8 $$F\left(\omega ,\tau \right)={\int }_{-\infty }^{\infty }f\left(t\right){{\psi }^{*}\left(t-\tau \right)e}^{-i\omega t}dt$$

$f\left(t\right)$ refers to the original signal, $\tau$ signifies the translation parameter, ${\psi }^{*}\left(t-\tau \right)$ denotes the window function (usually Hamming window), and when the window function uses a Gaussian function, the STFT is called a Gabor transform. Moreover, $e$ signifies the exponential operation, $i$ denotes the imaginary part, $t$ refers to time, and $\omega$ represents frequency. The STFT has a limitation in that its fixed time window results in a fixed time–frequency resolution [69].

#### WVD

The WVD is a classical method for time–frequency analysis that excels in handling non-stationary signals. Unlike STFT, the WVD remains unaffected by leakage effects. This distribution represents a secondary energy density, derived by correlating the signal with time and frequency translations along with their complex conjugates. The instantaneous autocorrelation function of the signal $f(t)$ is as follows:

9 $${R}_{f}\left(t,\tau \right)=f\left(t+\frac{\tau }{2}\right){f}^{*}\left(t+\frac{\tau }{2}\right)$$

$t$ refers to time, $\tau$ signifies the time-lag correlation coefficient, $*$ denotes the complex conjugate, and the WVD of $f(t)$ refers to the Fourier transform of ${R}_{f}\left(t,\tau \right)$ about $\tau$.

10 $$W\left(t,\omega \right)={\int }_{-\infty }^{+\infty }{R}_{f}\left(t,\tau \right){e}^{-j\omega \tau }d\tau$$

$e$ signifies the exponential operation, $j$ denotes the imaginary part, and $\omega$ represents frequency.

The WVD has a series of good properties such as conjugate symmetry, time-marginal properties, frequency-marginal properties, and energy distribution properties. However, when the signals have multiple frequency components, the WVD is affected by cross terms, that is, it is easily affected by noise [70].

#### WT

The WT overcomes the time–frequency resolution limitation observed in the STFT algorithm. This is achieved by introducing varying time–frequency resolutions in the outcomes through the translation and scaling of wavelets. Wavelets:

11 $${\psi }_{a,b}\left(t\right)=\left(\frac{1}{\sqrt{a}}\right)\psi \left(\frac{t-b}{a}\right)$$

$\psi \left(t\right)$ refers to the mother wavelet, ${\psi }_{a,b}\left(t\right)$ signifies the sub-wavelet, $a,b$ refer to the modulation and translation parameters, respectively, and the WT of the signal $f(t)$ is as follows:

12 $${W}_{\psi }f\left(a,b\right)=\frac{1}{\sqrt{\left|a\right|}}{\int }_{-\infty }^{\infty }f\left(t\right){\psi }^{*}\left(\frac{t-b}{a}\right)dt$$

Now, there are many optional mother wavelet functions (such as morse, morlet, db, and Harr), which include discrete and continuous wavelets. For EEG signal analysis, discrete wavelets are commonly used for signal decomposition, and continuous wavelets are commonly used for signal presentation. Thus far, although continuous wavelets cause massive data redundancy, they have been the preferred time–frequency analysis method, with more accurate and smooth time–frequency representation [69].

#### EMD

EMD is a self-adaptive multiresolution technique that decomposes the original signals into components of different resolutions and can analyze non-linear and non-smooth signals [71]. EMD decomposes the input signals into several intrinsic mode functions (IMFs) and a residual:

13 $$I\left(n\right)=\sum_{m=1}^{M}{IMF}_{M}\left(n\right)+{Res}_{M}\left(n\right)$$

$I\left(n\right)$ refers to a multi-component signal, ${IMF}_{M}\left(n\right)$ signifies the Mth IMF, and ${Res}_{M}\left(n\right)$ denotes the corresponding residual intrinsic modes. The IMF components usually extract time–frequency features using the Hilbert spectral analysis. EMD is characterized by self-adaptability and high efficiency. However, it may exhibit aliasing effects due to the presence of IMFs that contain significantly different characteristic time scales or when similar characteristic time scales are dispersed across different IMFs. EMD does not rely on the primary function of the fixed frequency, so the time–frequency results obtained by EMD are affected by Gibs; however, its positioning performance to the frequency is poor [72].

### Connectivity analyses

Connectivity analysis of EEG is an analytical method that has gained much attention in recent years and is fundamental to research on brain networks and connectivity. Connectivity analysis includes multiple types, such as signal morphology-based, signal phase-based, statistics-based, and information-based analyses. Different correlation analysis methods are oriented to different principles and the obtained results express different characteristics. Hence, it is important to accurately choose suitable connectivity analysis methods during brain network research. We introduce several common connectivity analysis methods here. Table 3 summarized some articles that explored various aspects of EEG signal analysis, including analysis for different severities of obstructive sleep apnea, synchrony measures for early Alzheimer’s disease diagnosis, correlation between EEG abnormalities and symptoms of autism spectrum disorder, EEG channel correlation for emotion recognition, quantitative EEG in ischemic stroke correlation with functional status, variability of EEG functional connectivity in young attention deficit hyperactivity disorder subjects, and the identification of causal relationships between EEG activity and intracranial pressure changes in neurocritical care patients, among other topics [73–106].

**Table 3:** Applications of correlation analyses

References	Data type	Subjects	Method	Disease/state	Application	Effect evaluation
Kang et al. [73]	EEG	SHHS dataset	CORR	Sleep stages	Analyzed the severity of symptoms in patients with OSA using the CORR method	Variations in microstructures were identified between the PSG-derived sleep EEG of non-OSA participants and those with varying severities of OSA in this study
Dauwels et al. [74]	EEG	MCI (*n* = 25) Normal (*n* = 56)	CORR	AD	Employed CORR to assess early symptoms of AD	Stochastic event synchrony was proposed as a feature to differentiate MCI patients from age-matched controls, achieving a leave-one-out classification rate of 83%, as reported in this article
Yasuhara [75]	EEG	Autistic children (*n* = 1014)	CORR	ASD	Used CORR to analyze the relationship between EEG abnormalities and ASD	The article suggested a correlation between ASD and dysfunction in the mirror neuron system
Islam et al. [76]	EEG	Normal (males = 16, females = 16)	CORR	Emotion	Integrated the CORR method with a CNN to identify emotions	Maximum accuracies of 78.22% on valence and 74.92% on arousal were attained using the internationally authorized DEAP dataset in this study
Sheorajpanday et al. [77]	EEG	Stroke (*n* = 110)	CORR	Stroke	Investigated the correlation between the EEG symmetry index and the Rankin scale and determined the prognostic value of EEG signals in the diagnosis of stroke	Prognostic value for disability, dependency, and death after 6 months in the subacute setting of ischemic stroke was attributed to EEG, according to this article
Alba et al. [78]	EEG	ADHD (*n* = 10) Normal (*n* = 12)	COH	ADHD	Adopted COH to analyze the functional connectivity of EEG in patients with ADHD under different resting states	Global connectivity of each region and its temporal variability were posited as better reflections of the underlying neural dysfunctions producing ADHD in this article
Carrasco-Gomez et al. [79]	EEG	Postanoxic coma (*n* = 594)	COH	Postanoxic coma	Assessed EEG functional connectivity in the context of post-anoxic coma through COH	The best non-coupling-based model, using data at 12 h and 48 h, achieved a sensitivity of 32% at 100% specificity, as claimed in this article
Barry et al. [80]	EEG	Normal (boys = 40, girls = 40)	COH	Developmental trends in normal children	Employed COH to analyze brain development in normal children of different ages and genders	This article asserted that EEG coherences in normal children aged 8 to 12 systematically develop with age
Locatelli et al. [81]	EEG	AD (*n* = 10) Normal (*n* = 10)	COH	AD	Analyzed the EEG signal characteristics of AD	Alpha coherence decrease was linked to alterations in cortico-cortical connections, while delta coherence increase was associated with the lack of influence of subcortical cholinergic structures on cortical electrical activity, as claimed in this article
Coben et al. [82]	EEG	ASD (*n* = 20) Normal (*n* = 20)	COH	ASD	Found neural underconnectivity in patients with ASD through COH, which is consistent with the results of other methods	Dysfunctional integration of frontal and posterior brain regions, along with a pattern of neural underconnectivity, was suggested in autistic subjects, as reported in this article
Catarino et al. [83]	EEG	ASC (*n* = 15) Normal (*n* = 15)	WTC	ASC	Probed task-related functional connections in the setting of the autism spectrum using the WTC algorithm	Impairment in task differentiation in individuals with ASC relative to typically developing individuals was reflected in this article
Omidvarnia et al. [84]	EEG	Epilepsy (*n* = 7)	WTC	Epilepsy	Discussed whether there was a direct correlation between EEG and regional hemodynamic brain connectivity changes in focal epilepsy	A strong time-varying relationship between local fMRI connectivity and interictal EEG power in focal epilepsy was claimed in this article
Khan et al. [85]	EEG	MDD (*n* = 30) Normal (*n* = 30)	WTC	MDD	Studied the diagnosis of depression using the WTC approach	An accuracy of 98.1%, sensitivity of 98.0%, and specificity of 98.2% were achieved in this article, with another method yielding 100% accuracy, sensitivity, and specificity
Sankari et al. [86]	EEG	AD (*n* = 20)	WTC	AD	Utilized the WTC method to explore the diagnosis of AD	WTC was proposed as a powerful tool to differentiate between healthy older individuals and probable AD patients in this article
Briels et al. [87]	EEG	SCD (*n* = 399) AD (*n* = 410)	PLV/PLI	AD	Analyzed the reproducibility of EEG functional connections in AD using PLV/PLI	In alpha/beta bands and PLI and wPLI in the theta band were highlighted for providing valid insights into disease-associated changes, correlating with disease severity, as indicated in this study
Olejarczyk et al. [88]	EEG	SZ (males = 7, females = 7) Normal (males = 7, females = 7)	PLV/PLI	SZ	Assessed brain connectivity in patients with SZ using PLV/PLI	Comparing different connectivity measures using graph-based indices for each frequency band separately was suggested as a useful tool in the study of connectivity disorders, such as SZ
Wang et al. [89]	EEG	DEAP dataset Normal (males = 7, females = 8)	PLV/PLI	Emotion	Explored the dynamics of rich-club structures in the brain during emotional changes, utilizing dynamic PLV brain networks and ReliefF algorithm to derive emotionally relevant features for accurate emotion recognition	Rich-club composition with subtle temporal variations was revealed, emphasizing the importance of small-scale structure connections in distinguishing emotions, achieving high accuracy (86.11% and 87.92%) in valence dimension validation on DEAP and SEED datasets
Huang et al. [90]	EEG	CAP dataset	PLV/PLI	Sleep stages	Highlighted the importance of exploring global information exchange between brain regions for improved sleep evaluation and disease diagnosis	High classification accuracy (96.91% intra-subject, 96.14% inter-subject) in sleep stage classification surpassed the performance of decision-level and hybrid fusion methods in this study
Zuchowicz et al. [91]	EEG	MDD (*n* = 8) BP (*n* = 10)	PLV/PLI	MDD	Explored the impact of repeated transcranial magnetic stimulation on patients with depression through the PLV/PLI approach	PLV analysis was indicated as a potential indicator of the response to depression treatment, enhancing therapy effectiveness in this research
Chen et al. [92]	EEG	ADHD (girls = 9) Normal (*n* = 51)	MI	ADHD	Adopted MI to extract the brain network of children with ADHD	A convincing performance with an accuracy of 94.67% regarding the test data was achieved in this article
Aydin et al. [93]	EEG	Normal	MI	Sleep stages	Analyzed the EEG of insomnia patients using MI	The level of cortical hemispheric connectivity was claimed to be strongly associated with sleep disorders in this article
Piho et al. [94]	EEG	DEAP dataset MAHNOB dataset	MI	Emotion	Determined emotion recognition features through MI	Significant improvement in emotion recognition accuracy was demonstrated in experimental results on publicly available datasets, as claimed in this article
Hassan et al. [95]	EEG	Bonn dataset CHB-MIT dataset	MI	Epilepsy	Applied MI to identify individual features for epileptic seizure detection	Significant performance improvement compared to recent state-of-the-art methods was reported in this article
Yin et al. [96]	EEG	SZ (positive = 14, negative = 14) Normal (*n* = 14)	MI	SZ	Analyzed brain functional connectivity in patients with SZ using the MI approach	Information interactions in SZ patients were claimed to be fewer than in normal controls, with positive SZ exhibiting more interactions than negative SZ, along with slower and less efficient information transfer between brain regions, according to this article
Sanz-García et al. [97]	EEG	SAH (*n* = 21)	GC	Subarachnoid hemorrhage	Used the GC algorithm to determine the causal relationship between EEG activity and changes in ICP in neurocritical care patients	A significant GC statistic from EEG activity to ICP was found during 37.88% of the analyzed time, with typical lags of 25–50 s between them, as reported in this article
de Tommaso et al. [98]	EEG	Migraine (males = 3, females = 28)	GC	Migraine	Adopted the GC algorithm to explore the functional connectivity of EEG signals in migraine patients responding to laser stimulation	Brain network analysis was suggested to aid in understanding subtle changes in pain processing under laser stimuli in migraine patients in this article
Nicolaou et al. [99]	EEG	Normal (males = 21)	GC	Anesthetized	Utilized the GC algorithm to distinguish between “awake” and “anesthetized” states	Features derived from GC estimates resulted in the classification of awake and "anesthetized" states in 21 patients with maximum average accuracies of 0.98 and 0.95, respectively, according to this article
Nicolaou et al. [100]	EEG	Normal (males = 21)	GC	Anesthetized	Utilized the GC algorithm to distinguish between “awake” and “anesthetized” states	The methodology of GC analysis of EEG data was claimed to carry implications for integrated information and causal density theories of consciousness in this article
Barrett et al. [101]	EEG	Normal (*n* = 7)	GC	Anesthetized	Investigated propofol-induced anesthesia using the GC algorithm	Significant increases in bidirectional GC during loss of consciousness, especially in the beta and gamma frequency ranges, were claimed in this article
Coben et al. [102]	EEG	Epilepsy (*n* = 2)	GC	Seizure location	Analyzed brain functional connectivity in epilepsy through the GC algorithm	Hypercoupling near the seizure foci and low causality across nearby and associated neuronal pathways were suggested in this article
Chen et al. [103]	EEG	MCI (*n* = 46) AD (*n* = 43)	MCI and AD	CFC	Analyzed resting state EEG in patients with MCI and AD using CFC	Alterations in theta-gamma coupling in the temporal lobe were claimed to become progressively obvious during disease progression, serving as a valuable indicator of MCI and AD pathology, as suggested in this article
Lynn et al. [104]	EEG	Not reported	SZ	CFC	Analyzed the working memory of schizophrenic patients using CFC	Formal testing of theta-gamma interaction was proposed as imperative in this article
Papadaniil et al. [105]	EEG	Normal (males = 14)	Auditory Perception	CFC	Used CFC to study auditory perception tasks	Stronger coupling in the delta band, closely linked to sensory processing, was observed and claimed in this article
Park et al. [106]	EEG	Normal	visual memories	CFD	Used CFD to study the formation of visual memory	Encoding of visual information reflecting a state determined by the interaction between alpha and gamma activity was asserted in this article

*AD* Alzheimer’s disease, *ADHD* attention deficit hyperactivity disorder, *ASC* autism spectrum condition, *ASD* autism spectrum disorder, *CFC* cross-frequency coupling, *CFD* cross-frequency directionality, *CHB-MIT* Children’s Hospital Boston and the Massachusetts Institute of Technology, *COH* coherence, *CORR* correlation, *DEAP* database for emotion analysis using physiological signals, *EEG* electroencephalography, *fMRI* functional magnetic resonance imaging, *GC* granger causality, *ICP* intracranial pressure, *MAHNOB* Multimodal Database for Affect Recognition and Implicit Tagging, *MCI* mild cognitive impairment, *MDD* major depressive disorder, *MI* mutual information, *OSA* obstructive sleep apnea, *PLV/PLI* phase lag value/index, *PSG* polysomnography, *SEED* Shanghai Jiao Tong University emotion EEG dataset, *SHHS* sleep heart health study, *SZ* schizophrenia, *wPLI* weighted phase lag index, *WTC* wavelet coherence

#### Correlation (CORR)

CORR measures the similarity between two signals by calculating the variance of signals [107]. The CORR for each given frequency is as follows:

14 $$Corr\left(x\right)=\frac{{C}_{AB}\left(x\right)}{\left({C}_{AA}\left(x\right){C}_{BB}\left(x\right)\right)}$$

${C}_{AB}\left(x\right)$ represents the cross-covariance between signal $A$ and signal $B$, and ${C}_{AA}\left(x\right)$ and ${C}_{BB}\left(x\right)$ refer to the auto-covariance of signal $A$ and signal $B$, respectively. CORR is sensitive to both phase and polarity.

#### Coherence (COH)

COH measures the similarity between two signals by calculating the power spectral density [108]. The COH for each given frequency is:

15 $$COH\left(x\right)=\frac{{\left|{S}_{AB}\left(x\right)\right|}^{2}}{\left({S}_{AA}\left(x\right){S}_{BB}\left(x\right)\right)}$$

${S}_{AB}\left(x\right)$ represents the cross-spectrum between signal $A$ and signal $B$, and ${S}_{AA}\left(x\right)$ and ${S}_{BB}\left(x\right)$ refer to the auto-covariance of signal $A$ and signal $B$, respectively. Because COH is calculated through cross-spectrum and auto-spectrum, it is very sensitive to the phase changes of the signal but is little affected by energy changes.

#### Wavelet coherence (WTC)

WTC can represent the time-varying relationships between different signals in the time–frequency domain by producing different time–frequency resolutions through wavelet translation and dilation [109].

The WT of signal $x$ is a function of time and frequency, defined as the convolution of an input with a wavelet family $\theta (u)$:

16 $${W}_{x}\left(t,f\right)={\int }_{-\infty }^{\infty }x\left(u\right){\theta }_{t,f}^{*}\left(u\right)du$$

With given input signals $x$ and $y$, the wavelet cross-spectrum around time $t$ and frequency $f$ can be derived through the WT of $x$ and $y$:

17 $$C{W}_{xy}\left(t,f\right)={\int }_{t-\delta /2}^{t+\delta /2}{W}_{x}\left(\tau ,f\right){W}_{y}^{*}\left(\tau ,f\right)d\tau$$

Here, $*$ represents the complex conjugate, and $\delta$ denotes the scalar dependent on the frequency. The WTC of time $t$ and frequency $f$ is represented by $C{W}_{xx}\left(t,f\right)$, and $C{W}_{yy}\left(t,f\right)$ refers to the Fourier transform of the autocorrelation function of signal $x$ and signal $y$. WTC can view the phase correlation between signals on the time spectrum and reduce the interference of energy.

#### Phase lag value/index (PLV/PLI)

PLV and PLI are commonly applied to acquire the strength of phase synchronization [110]. The instantaneous phase of signal $x(t)$ is generated using the following formula:

18 $${\varnothing }_{x}\left(t\right)=arctan\frac{\widetilde{x}\left(t\right)}{x\left(t\right)}$$

Here, $\widetilde{x}\left(t\right)$ signifies the Hilbert transform of $x\left(t\right)$, defined as follows:

19 $$\widetilde{x}\left(t\right)=\frac{1}{\pi }PV{\int }_{-\infty }^{\infty }\frac{x(\tau )}{t-\tau }d\tau$$

PV refers to the Cauchy principal value. The PLV of two signals is defined as follows:

20 $$PLV=\left|\frac{1}{N}\sum_{j=0}^{N-1}{\mathrm{e}}^{\left(j\left({\varnothing }_{x}\left(j\Delta t\right)\right)-{\varnothing }_{y}\left(j\Delta t\right)\right)}\right|$$

Here, $\Delta t$ denotes the sampling period, $N$ represents the number of samples per signal, $j$ refers to the imaginary part, and $e$ signifies the exponent. PLV signifies phase synchronization, with values ranging from 0 to 1. A value of 0 indicates a lack of synchronization, while 1 represents strict phase synchronization. On the other hand, PLI characterizes the asymmetry in the phase difference distribution between two signals. It is computed based on the relative phase difference between the two signals:

21 $$PLI=\left|E[sign(\Delta \varnothing (t))]\right|$$

$E$ represents expectation, the result value is located within the interval [0, 1], and a higher value indicates a higher phase synchronization.

#### Mutual information (MI)

MI is designed based on information theory, which presents how one signal provides information for another signal [109]. $P({x}_{j})$ and $P({y}_{j})$ are the probability distributions of signal $X=\{{x}_{j}\}$ and signal $Y=\{{y}_{j}\}$, respectively. The entropy of $X$ and $Y$ is defined as follows:

22 $$H\left(X\right)=-\sum_{j=1}^{N}P\left({x}_{j}\right)log\left(P\left({x}_{j}\right)\right)$$

23 $$H\left(Y\right)=-\sum_{j=1}^{N}P\left({y}_{j}\right)log\left(P\left({y}_{j}\right)\right)$$

*N* signifies the window length. $H\left(\left.Y\right|X\right)$ and $H\left(X,Y\right)$ refer to the conditional entropy and joint entropy between $X$ and $Y$, respectively, which are defined as:

24 $$H\left(X,Y\right)=-{E}_{X}\left[{E}_{Y}\left[logP\left(X,Y\right)\right]\right]$$

25 $$H\left(\left.Y\right|X\right)=-{E}_{X}\left[{E}_{Y}\left[logP\left(\left.Y\right|X\right)\right]\right]$$

Here, *E* denotes the expected value function. The MI of signal *X* and signal *Y* is calculated as follows:

26 $$MI\left(X,Y\right)=H\left(X\right)+H\left(Y\right)-H\left(X,Y\right)=H\left(Y\right)-H\left(\left.Y\right|X\right)$$

MI can simultaneously detect the linear and nonlinear correlations between two signals, but it requires mass data.

#### Granger causality (GC)

GC is a linear vector AR model based on random time-series data, which can estimate effective interactions from time-series data [111]. For this method, if the past value of the signal ${X}_{1}(t)$ contains information that contributes to the prediction of ${X}_{2}(t)$, which exceeds the information contained only in the past value of *Y*, the signal ${X}_{1}(t)$ “Granger causes” the signal ${X}_{2}(t)$. Therefore, the bivariate AR model is as follows:

27 $${X}_{1}\left(t\right)=\sum_{j=1}^{p}{A}_{11,j}{X}_{1}\left(t-j\right)+\sum_{j=1}^{p}{A}_{12,j}{X}_{2}\left(t-j\right)+{E}_{1}\left(t\right)$$

28 $${X}_{2}\left(t\right)=\sum_{j=1}^{p}{A}_{21,j}{X}_{1}\left(t-j\right)+\sum_{j=1}^{p}{A}_{22,j}{X}_{2}\left(t-j\right)+{E}_{2}\left(t\right)$$

$p$ refers to the maximum number of delayed observations, $j$ denotes the lag coefficient, the matrix $A$ represents the contribution of each delayed observation to the predicted signal value, and ${E}_{1}\left(t\right)$, ${E}_{2}\left(t\right)$ signify the residual of each time series. GC can only provide information on the linear characteristics of the signal and cannot analyze nonlinear situations.

#### Cross-frequency analysis (CFA)

CFA is a kind of rapidly developing connectivity analysis method, which mainly includes cross-frequency coupling (CFC) and cross-frequency directionality (CFD).

CFC describes the interaction of brain oscillations across different frequency bands and manifests in four modes: phase-to-amplitude, power-to-power, phase-to-phase, and phase-to-frequency interactions. The Kullback–Leibler distance serves as an effective metric for quantifying CFC [112]. Notably, CFC holds significance in working memory processes [113]. According to the theta/gamma neural code hypothesis, conserved memory items are encoded through theta-nested gamma cycles in sensory regions, facilitating communication between different brain cortices during memory and sensory processes [114]. A study leveraging iEEG data in epilepsy patients, coupled with behavioral outcomes, underscore the association between theta/gamma CFC across diverse brain regions and working memory performance [115]. Key findings reveal the widespread distribution of theta/gamma phase amplitude coupling across the cortex, with increased coupling strength observed in more cognitively demanding working memory tasks [116].

CFD, measuring information flow direction between brain regions, involves the modulation of high-frequency signal amplitude by the phase of a low-frequency signal [117]. It relies on the phase slope index, quantifying the phase slope in the cross-spectrum of two signals [117]. CFD has proven valuable for inferring causal relationships and estimating signal delays [118]. Additionally, it has been employed in exploring information flow directions between distinct brain regions during various cognitive tasks [117].

### Source localization analysis

With the continuous development of EEG and MEG devices, the number of channels in scalp EEG or MEG has increased to over 100. Multi-channel and multi-location EEG/MEG signals have accelerated the development of EEG source localization.

First, structural MRI is often used as a prior in source localization analysis because it provides a high-resolution three-dimensional (3D) image of the brain’s anatomy. This image can be used to create a head model that accurately represents the geometry and conductivity of the brain and skull [119]. The head model is then used to calculate the forward solution, which describes how electrical activity generated by the brain is measured at the scalp [120]. By incorporating structural MRI information into the forward solution, the accuracy of the source localization can be improved.

Moreover, structural MRI has the potential to generate an accurate boundary element model of the head, facilitating the computation of the lead field matrix [121]. This matrix characterizes the propagation of electrical activity generated by the brain to the scalp electrodes [122]. The utilization of a realistic boundary element model enhances the precision of lead field matrix calculations, thereby improving the accuracy of source localization [123].

The source localization method can infer the intracranial discharge status of the brain through multi-channel signals from the scalp, human brain physical models, and finite element calculations. Source localization methods are commonly employed to localize functional areas and lesion areas, among others, under non-invasive conditions. Common source localization methods are introduced below. The articles in Table 4 explored EEG source localization techniques, including dipole analysis, beamforming approaches, and methods like low-resolution electromagnetic tomography (LORETA) and standardized LORETA (sLORETA), to study various conditions such as epilepsy, visual working memory tasks, auditory attention, depression, obsessive–compulsive disorder, pain perception, age-related hearing loss, and different neurological disorders, providing insights into the localization of brain activity in these contexts [124–151].

**Table 4:** Applications of source localization analyses

References	Data type	Subjects	Method	Disease/state	Application	Effect evaluation
Toole et al. [124]	EEG	Epilepsy (*n* = 9)	MNE	Seizure location	Investigated the specificity of epileptic patients using the minimum norm	HFA observed in tEEG was found to be localized to the surface of subject-specific cortical models, occurring predominantly at seizure onset, as per the assertions in this article
Galaris et al. [125]	EEG	Epilepsy (boys = 10, girls = 11)	MNE	Seizure location	Conducted an EEG source localization analysis during a visual working memory task in children with epilepsy using the minimum norm	The spatio-temporal patterns of differences between groups of epileptic and control children were claimed to be consistent across all three methods, according to this article
Lee et al. [126]	EEG/MEG	Normal (*n* = 1)	MNE	ERP location	Employed the minimum norm to study the source localization of auditory stimuli	Utilizing individual anatomical MRI data, this article asserted the possibility of establishing a relationship between sensor information and dipole activation on the cortex
Sperli et al. [127]	EEG	Epilepsy (males = 11, females = 19)	MNE	Seizure location	Examined the application of source localization algorithms in pediatric epilepsy using the minimum norm	The ESI was claimed to compare favorably to other imaging techniques, achieving a success rate of 90%, positioning it as a valuable tool for epilepsy surgery planning in children, as stated in this article
Plummer et al. [128]	EEG	Epilepsy (children = 8)	FOCUSS	Seizure location	Performed source localization analysis of EEG during seizures in patients with focal epilepsy using the FOCUSS algorithm	The clinical utility of routine work-up for unilateral BFEC and unilateral MTLE secondary to hippocampal sclerosis was demonstrated using distributed source modeling in this article
Wei et al. [129]	EEG	Epilepsy (*n* = 1)	FOCUSS	Seizure location	Combined the FOCUSS algorithm with the LORETA algorithm for epileptic focus localization	The article suggested the potential use of estimated source energy trends for predicting epileptic seizures, showcasing the algorithm’s application in both localization and prediction aspects
Ye et al. [130]	EEG	Normal (*n* = 2)	FOCUSS	ERP location	Reconstructed MRI images with the FOCUSS algorithm	The new algorithm’s successful application for synthetic data and in vivo brain imaging obtained by an under-sampled radial spin echo sequence was claimed in this article
Saletu et al. [131]	EEG	Depressed menopausal syndrome (females = 60) Menopausal syndrome (females = 30) Normal (females = 30)	LORETA	Pharmacotherapy of depression	Used the LORETA algorithm to study the effects of drugs on patients with depression before and after treatment	EEG activity in the theta band was claimed to be increased in anatomically meaningful patterns in patients, differing from the distribution in healthy individuals, according to this article
Clemens et al. [132]	EEG	Epilepsy (*n* = 40) Normal (*n* = 14)	LORETA	Seizure location	Applied spectral analysis and LORETA to investigate and localize the sources of spontaneous theta activity in patients with partial epilepsy, distinguishing between untreated and treated groups, as well as healthy individuals	Untreated partial epilepsy patients were reported to display bilateral theta maxima in specific brain areas, while treated patients showed increased theta activity across the scalp with shifting abnormality centers in certain areas, as revealed in this article
Kopřivová et al. [133]	EEG	OCD (*n* = 50) Normal (*n* = 50)	LORETA	OCD	Utilized sLORETA and normative ICA to assess intracortical EEG sources in 50 patients with OCD, revealing increased low-frequency activity in the medial frontal cortex compared to matched controls	Low-frequency power excess in the medial frontal cortex of OCD patients was indicated through sLORETA and group ICA methods, providing consistent evidence for medial frontal hyperactivation in OCD, as reported in this article
Shao et al. [134]	EEG	Normal (*n* = 26)	LORETA	Acute tonic pain	Conducted a brain source localization analysis of tonic cold pain with the LORETA algorithm	Changes in cortical source power across different frequency bands in multiple brain regions were demonstrated as potential electrocortical indices of acute tonic pain, correlating with subjective pain ratings, in this article
Loughrey et al. [135]	EEG	Normal (*n* = 14) Hearing loss (*n* = 44)	sLORETA	Hearing loss	Used the sLORETA method to study the relationship between age-related hearing loss and visual working memory	Greater activity in networks modulated by frontoparietal and temporal regions was indicated through sLORETA analyses in this article
Dubová et al. [136]	EEG	Normal (males = 5, females = 5)	sLORETA	Mirrored touch	Used the sLORETA method for the brain projection of mirrored touch	The summation of stimuli secured by interpersonal haptic contact modified by mirror illusion was claimed to activate brain areas integrating motor, sensory, and cognitive functions, as well as areas related to communication and understanding processes, including the mirror neuron system, according to this article
Liu et al. [137]	EEG	Vestibular migraine (females = 33) Normal (females = 20)	sLORETA	Vestibular migraine	Studied visual evoked potentials in patients with vestibular migraine using the sLORETA method	This article suggested that abnormalities in vestibular cortical fields might be a pathophysiological mechanism of vestibular migraine
Yoshinaga et al. [138]	EEG	Epilepsy (boys = 4, girls = 4)	Dipole	Panayiotopoulos syndrome	Analyzed EEG signals in patients with panayiotopoulos syndrome, a form of benign childhood partial epilepsy, using the dipole method	A potential pathogenetic link between panayiotopoulos syndrome and rolandic epilepsy was suggested in this article
Ebersole [139]	iEEG	Epilepsy (*n* = 10)	Dipole	Seizure location	Used dipole models for the non-invasive localization of epileptogenic foci	Patients with lateral temporal cortical seizures were claimed to have spikes and ictal activity modeled principally by radial dipoles, as reported in this article
Nakajima et al. [140]	EEG	Stroke (*n* = 1)	Dipole	Cerebral infarction	Employed the dipole method to track and analyze brain potentials in patients with stroke	The dipole equivalent of the slow wave was reported to be approximately located in the frontal part of the left cingulate gyrus in this article
Verhellen et al. [141]	EEG/iEEG	Epilepsy	Dipole	Seizure location	Explored the localization of refractory temporal lobe epilepsy through the dipole method	Dipole localizations and intracerebral fields recorded with depth electrodes were compared in this article
Ntolkeras et al. [142]	EEG/MEG/iEEG	Epilepsy (boys = 7, girls = 4)	Dipole	Seizure location	Conducted a comparison and validation analysis of epileptic patients before and after surgical resection using the dipole method	Magnetic and ESI dipole clustering was claimed to help localize the seizure onset zone and irritative zone, facilitating the prognostic assessment of MRI-negative patients with drug-resistant epilepsy
Knyazev et al. [143]	EEG	Normal (males = 19, females = 36)	Beamforming	Depression	Conducted a beamforming analysis on the EEG signals of depressed patients when they completed different tasks	Emotional circuits were asserted to be more strongly connected with DMN than TPN in this article
Neugebauer et al. [144]	EEG/MEG	Epilepsy (male = 1, female = 1)	Beamforming	Seizure location	Utilized the beamforming method to explore epileptogenic zones in focal cortical dysplasia	The beamformer was claimed to localize better than the standard dipole scan for appropriate regularization parameter choices in this article
Ward et al. [145]	EEG	Epilepsy (*n* = 4)	Beamforming	Seizure location	Analyzed deep epileptic form activity using beamforming techniques	The beamformer was demonstrated to enhance signals from deep foci, improving SNR and showing promise in the detection of epileptiform events in this article
Kouchaki et al. [146]	EEG	Normal (*n* = 17)	Beamforming	Brain fatigue	Employed the beamforming approach to explore brain changes from non-fatigued to fatigued states	The proposed MVB-based feature, applied to SVM classification, achieved a remarkable 97.06% accuracy in differentiating between non-fatigue and fatigue mental states, significantly outperforming conventional EEG features, as highlighted in this study
Vergallo et al. [147]	EEG	Simulated signals	Beamforming	Seizure location	Adopted the beamforming method to diagnose epilepsy	Simple geometry, simulations, and results demonstrating the performance of several algorithms were considered in this article
Ponomarev et al. [148]	EEG	ADHD (females = 46, males = 50) Normal (males = 167, females = 209)	CSD	ADHD	Analyzed EEG signals in patients with ADHD using CSD	The spectral power of local EEG activity isolated by gICA or CSD in fronto-central areas was suggested as a suitable marker for discriminating ADHD patients and healthy adults in this article
Stewart et al. [149]	EEG	Normal (males = 95, females = 211)	CSD	Major depressive disorder	Utilized CSD for analyzing resting-state and task-evoked prefrontal EEG asymmetry in patients with depression	CSD-transformed data was claimed to be a more robust indicator of trait frontal EEG asymmetry, according to this article
Grin-Yatsenko et al. [150]	EEG	SZ (males = 36, females = 12) Normal (males = 217, females = 286)	CSD	SZ	Employed the CSD method to analyze brain activity in schizophrenic patients	Differences in the delta and theta range were claimed to be described mainly by local components, and those in the beta range mostly by spatially widely distributed ones, in this article
Kamarajan et al. [151]	EEG	Alcoholics (males = 38) Normal (males = 38)	CSD	Alcohol	Probed alcohol dependence using the CSD approach	Decreased power and a weaker, more diffuse CSD in alcoholics were claimed to be due to dysfunctional neural reward circuitry, as suggested in this article

*ADHD* attention deficit hyperactivity disorder, *BFEC* benign focal epilepsy of childhood, *CSD* current source density, *DMN* default-mode network, *EEG* electroencephalography, *ERP* event-related potential, *ESI* electric source imaging, *FOCUSS* focal underdetermined system solution, *HFA* high-frequency activity, *ICA* independent component analysis, *iEEG* intracranial electroencephalography, *LORETA* low-resolution electromagnetic tomography, *MEG* magnetoencephalography, *MNE* minimum-norm estimates, *MRI* magnetic resonance imaging, *MTLE* mesial temporal lobe epilepsy, *MVB* minimum variance beamformer, *OCD* obsessive–compulsive disorder, *sLORETA* standardized low-resolution electromagnetic tomography, *SNR* signal-to-noise ratio, *SZ* schizophrenia, *SVM* support vector machine, *TPN* task-positive network, *tEEG* tripolar electroencephalography, *gICA* group independent component analysis

#### Minimum norm estimation

The minimum norm estimation method uses MEG for analysis and solves the current distribution by estimating the linear combination of the magnetometer lead field. ${L}_{i}$ signifies the vector field at the position $i$, so the output of the magnetometer is defined as follows:

29 $${B}_{i}\left(J\right)=\int {L}_{i}\left(r\right)J\left(r\right)dV$$

$J\left(r\right)$ denotes the conversion of various energy types into electrical energy, and the linear relationship among the magnetometer reading, current distribution, and lead field is expressed as:

30 $$B=LJ$$

Consequently, the shortest current vector needed to explain the magnetometer output is defined by multiplying the output vector $B$ by the pseudo-inverse of $L$:

31 $$\widehat{J}={L}^{+}B$$

Here, ${L}^{+}={L}^{T}{(L{L}^{T})}^{+}$ represents the Moore–Penrose generalized inverse, predicting minimum norm solutions for pure signals, noise-contaminated signals, and smoothed noise signals. Due to the harmonic nature of the minimum norm solution, the method faces challenges in resolving deep source localization within the outermost cortex, leading to localization errors [152].

#### Focal underdetermined system solution (FOCUSS)

FOCUSS is a high-resolution non-parametric technique that allocates current to each element within a predetermined reconstruction region using a forward model [153]. The weighted minimum norm method is used to perform mathematical calculations in the recursive steps in focusing. The calculation formula for the unknown current element $I$ is as follows:

32 $$I=W{\left(GW\right)}^{+}B=W{W}^{T}{G}^{T}{\left(GW{W}^{T}{G}^{T}\right)}^{-1}B$$

Here, $W$ is an $n \times n$ matrix that refers to a constraint on the results to strengthen some elements in $I$, $B$ denotes the measured value of the radial magnetic field, and $G$ signifies the spatial weight of the element:

33 $${W}_{k}=\left[\begin{array}{ccccc}{I}_{1_{k-1}}&\quad \empty &\quad 0\\ \qquad \quad \ddots&\quad \empty &\quad \empty \\ \empty &\quad {I}_{i_{k-1}}&\quad \empty \\ \empty &\qquad \quad \ddots&\quad \empty\\ 0 &\quad\empty&\quad {I}_{n_{k-1}}\end{array}\right]$$

${I}_{ik-1}$ represents the $i$th element of vector $I$ in the $(k-1)$ iteration, and $k$ signifies the index of the iteration step. By continuously constructing $W$ and calculating the weighted minimum norm, the model results are converged, but the computation time of FOCUSS is longer than that of other algorithms.

#### LORETA

LORETA is an innovative method in the high temporal resolution neuroimaging field that allows for the 3D reconstruction of the EEG activity distribution [154]. A head model is used for LORETA, and the intensity and direction of electrical activity at each point determine the electromagnetic field measured on the scalp. It is defined as:

34 $$\underset{J}{\mathrm{min}}{F}_{W}$$

with

35 $${F}_{W}={\Vert \Phi -KKJ\Vert }^{2}+\alpha {J}^{T}WJ$$

In the above equation, $\Phi$ represents a vector of potential difference, $K$ denotes the lead field matrix of the volume, $J$ signifies the current density, $W$ denotes the discrete Laplace operator in the square space, and $\alpha$ refers to the Tikhonov regularization parameter.

36 $${\widehat{J}}_{W}={T}_{W}\mathrm{\varnothing }$$

The ${T}_{W}$ value can be calculated using the following formula:

37 $${T}_{W}={W}^{-1}{K}^{T}{\left(K{W}^{-1}{K}^{T}+\alpha H\right)}^{+}$$

$H$ denotes the mean reference operator, which is realized using the discrete spatial Laplacian operator, so the spatial resolution of LORETA is relatively low.

sLORETA is also a common and popular source localization method. sLORETA incorporates additional assumptions regarding the smoothing and weighting of the values [155]. An advantage of sLORETA is that it has “guaranteed accuracy” in the presence of a single dipole, while LORETA does not [155]. sLORETA has been used in various studies to estimate the sources of EEG signals in the brain [156]. For example, sLORETA has been used to study the neural correlates of cognitive processes such as attention, memory, and language [156]. sLORETA has also been used to study the neural correlates of various disorders such as depression, schizophrenia, and Alzheimer’s disease [156].

#### Dipole

The dipole method can predict the electric field generated by a theoretical dipole in the brain using dipole property-related principles [157]. Location and orientation are two parameters of the dipole model, with the location indicating the position of active region within the brain in this model and the orientation indicating the arrangement of brain cells in the active region.

The six parameters of a dipole source consist of three coordinates in ${r}_{d}\in {R}^{3\times 1}$ and three dipole components in $d=({d}_{x},{d}_{y},{d}_{x})\in {R}^{3\times 1}$ (equivalently two orientation angles and an intensity parameter). For each dipole position ${r}_{d}$ within the head, the relation between $d$ and the potential measured at the $m$th electrode ${V}_{mod}\in {R}^{m\times 1}$ can be written as:

38 $${V}_{mod}=L\left({r}_{d}\right)d$$

The matrix $L\in {R}^{m\times 3}$ is a lead field matrix, determined by dipole position, electrode position, and head geometry.

A more realistically shaped head model is often required for patient EEG data analysis, but in this case, boundary element methods or numerical methods such as the finite-difference method are needed to compute the lead field matrix.

#### Beamforming

Beamforming is a spatial filtering technique for signals measured by discrete sensors [158]. Beamforming refocuses the signals captured on the scalp to their original locations by finding the weights of each location in the source space, thus minimizing the variance of the current dipole at each location. It is often desirable to extract signals from a small region of the brain that is modeled by dipoles at the location ${r}_{d}$ with a specific orientation. With a given dipole and its components, the potential distribution is defined as follows:

39 $$c= L\left({r}_{d}\right)d$$

40 $${w}^{T}c=1$$

${r}_{d}$ denotes the dipole coordinate, $d$ represents the dipole component, $L$ signifies the lead field matrix, and $w$ refers to the weight vector.

The output variance or output power of a beamformer is calculated as follows:

41 $$\varepsilon \left\{{\left|y\left(k\right)\right|}^{2}\right\},k=-\infty ,\dots ,\infty$$

$y(k)$ represents the output and $\varepsilon \left\{{|\cdot |}^{2}\right\}$ denotes the expected value of its parameter. The results are constrained with different restrictions.

#### Current source density (CSD)

CSD calculates an estimate of the current projected radially from the underlying neuronal tissue at a given surface location to the skull and scalp and calculates a spatially weighted sum of the potential gradients pointing to that location from some or all of the recorded locations.

CSD estimates:

42 $$C\left(E\right)=\sum_{i=1}^{N}{c}_{i}h\left(\mathrm{cos}\left(E,{E}_{i}\right)\right)$$

Here, $C\left(E\right)$ denotes the current density value at any point $E$ on the sphere surface, ${c}_{i}$ refers to a constant to express an $i$ surface potential set, and $\mathrm{cos}\left(E,{E}_{i}\right)$ refers to the cosine of the angle between the surface point $E$ and the electrode projection ${E}_{i}$. The function $h\left(x\right)$ is defined as the sum of the grades:

43 $$h\left(x\right)=\frac{1}{4\pi }\sum_{n-1}^{\infty }\frac{2n+1}{{n}^{m-1}{\left(n+1\right)}^{m-1}}{P}_{n}\left(x\right)$$

Here, $m$ is a constant greater than 1 and ${P}_{n}$ is the $n$th Legendre polynomial, defined as follows:

44 $$\Delta {P}_{n}=-n\left(n+1\right){P}_{n}$$

CSD does not necessitate reference information but is susceptible to noise. CSD is a source localization method designed for scalp EEG, treating the entire head as a conductor with equal conductivity. It concentrates signals from multiple EEG channels to their respective channels by adjusting the parameters. This method cannot focus EEG signals to the intracranial region. However, its arithmetic is simple and fast, and it is still used in some EEG analyses for scalp localization without the assistance of brain models [159].

### Machine learning

Machine learning is a very popular class of signal processing methods currently applied in the medical field [160], and with the rapid development of deep learning, machine learning methods in EEG analyses have gained attention. Machine learning methods are commonly used for classification and regression problems in EEG analyses and have yielded substantial results in disease research [161]. The studies in Table 5 utilized various machine learning and signal processing techniques, including common spatial pattern (CSP), deep learning, wavelet analysis, support vector machine (SVM), convolutional neural network (CNN), recurrent neural network (RNN), and long short-term memory (LSTM) network, to address diverse applications such as seizure detection, diagnosis of neurological disorders (autism, schizophrenia, Parkinson’s disease), mental fatigue measurement, and emotion recognition using EEG signals [162–185].

**Table 5:** Applications of machine learning methods

References	Data type	Subjects	Method	Disease/state	Application	Effect evaluation
Qaraqe et al. [162]	EEG	CHB-MIT dataset	CSP	Epilepsy	Utilized the CSP approach for seizure detection	A sensitivity of 100%, a detection latency of 7.28 s, and a false alarm rate of 1.2 per hour were successfully attained in this article
Dissanayake et al. [163]	EEG	CHB-MIT dataset	CSP	Epilepsy	Adopted the CSP algorithm for patient-independent seizure prediction	Accuracy achievements of 88.81% and 91.54% were reported in this article
Liu et al. [164]	EEG	BCI competition III-4a BCI competition IV-2a strokes (*n* = 5)	CSP	Stroke	Investigated the rehabilitation of stroke patients using the CSP algorithm	High accuracies were achieved in comparison with seven state-of-the-art approaches, as highlighted in this article
Alturki et al. [165]	EEG	Normal (males = 10) ASD (males = 6, females = 3) CHB-MIT dataset	CSP	Epilepsy and ASD	Applied the CSP algorithm for the diagnosis of epilepsy and autism	Accuracy rates of approximately 98.46% for diagnosing ASD and 98.62% for epilepsy were achieved in this article
Jamal et al. [166]	EEG	ASD (*n* = 12) Normal (*n* = 12)	LDA	ASD	Carried out LDA to classify ASD	Leave-one-out cross-validation of the classification algorithm resulted in a best performance of 94.7% accuracy, with corresponding sensitivity and specificity values of 85.7% and 100%, as reported in this article
Jeong et al. [167]	EEG	PDD (*n* = 26) AD (*n* = 26) Normal (*n* = 26)	LDA	PDD and AD	Applied LDA to distinguish between PD-related dementia and AD	A maximum performance of 80.19% accuracy was achieved using LDA with WC in this article
Boostani et al. [168]	EEG	SZ (males = 13) Normal (males = 18)	LDA	SZ	Adopted LDA in the diagnosis of SZ	Accuracies of 87.51%, 85.36%, and 85.41% were achieved for BDLDA, LDA, and Adaboost, respectively, in this article
Rajaguru et al. [169]	EEG	Epilepsy (*n* = 20)	LDA	Epilepsy	Used the LDA approach for the classification of epilepsy	When the dB2 and dB4 wavelets were classified with LDA, average classification accuracies of 95.83% and 95.03% were obtained, as claimed in this article
Kang et al. [170]	EEG	ASD (boys = 39, girls = 10) TD (boys = 36, girls = 12)	SVM	ASD	Employed the SVM method to identify children with ASD	Combining two types of data resulted in a maximum accuracy of 85.44%, with AUC = 0.93 when 32 features were selected in this article
Fu et al. [171]	EEG	Bonn dataset	SVM	Epilepsy	Adopted the SVM approach for the classification of epilepsy	A 99.125% accuracy of the algorithm with the theta rhythm of EEG signals was achieved in this article
Shen et al. [172]	EEG	Normal (*n* = 10)	SVM	Mental fatigue measurement	Utilized the SVM method for mental fatigue measurement	An accuracy of 87.2% for the probabilistic multi-class SVM compared to 85.4% using the standard multi-class SVM was reported. With confidence estimates aggregation, the accuracy increased to 91.2%
Liu et al. [173]	iEEG	Epilepsy (*n* = 21)	SVM	Epilepsy	Performed seizure detection in long-term EEG using the SVM method	A sensitivity of 94.46%, specificity of 95.26%, and a false detection rate of 0.58/h for seizure detection in long-term iEEG were achieved in this article
Zhou et al. [174]	EEG	CHB-MIT dataset	CNN	Epilepsy	Detected seizures through CNN models	The article achieved a convincing performance with an accuracy of 94.67% on the test data
Hassan et al. [175]	EEG	Bonn dataset	CNN	Epilepsy	Detected epilepsy through the 1D-CNN approach	Using frequency domain signals, average accuracies of 96.7%, 95.4%, and 92.3% for the three experiments were achieved in the Freiburg database, while average accuracies for detection in the CHB-MIT database were 95.6%, 97.5%, and 93% for the three experiments in this article
Hassan et al. [176]	EEG	SZ (*n* = 14) Normal (*n* = 14)	CNN	SZ	Detected SZ through the 1D-CNN approach	The article effectively predicted two, three, four, and five classes with accuracies of 100%, 99%, 94.6%, and 94%, respectively, for the Bonn dataset and 98% for the CHB-MIT dataset
Dong et al. [177]	EEG	ASD (children = 86) Normal (children = 89)	CNN	ASD	Applied the CNN method for the assessment of ASD in children	Accuracies of 90% and 98% were achieved for subject-based and non-subject-based testing, respectively, in this article
Aliyu et al. [178]	EEG	Bonn dataset	CNN	Epilepsy	Detected epileptic EEG signals using CNN	The method was claimed to outperform its counterparts, achieving individual/sample accuracy of 92.63%/83.23%, as reported in this article
Lee et al. [179]	EEG	PD (*n* = 20) Normal (*n* = 20)	RNN	PD	Combined CNN with RNN for the identification of PD	An accuracy of 99.2%, precision of 98.9%, and recall of 99.4% in differentiating PD from healthy controls were achieved in this article
Sarkar et al. [180]	EEG	Normal (male = 1, female = 1)	RNN	Mental depression	Detected mental depression through RNN	The article achieved the highest accuracies of 97.50% in the training set and 96.50% in the test set
Mishra et al. [181]	EEG	Sleep-EDF dataset	RNN	Sleep stages	Employed CNN and RNN for sleep stage classification	Efficient classification performance in sleep stage N1, as well as improvement in subsequent stages of sleep, was reported in this article
Michielli et al. [182]	EEG	Normal (*n* = 10)	LSTM	Sleep stages	Used LSTM for the classification of different sleep stages	The overall percentage of correct classifications for five sleep stages was found to be 86.7% in this article
Hu et al. [183]	EEG	CHB-MIT dataset	LSTM	Epilepsy	Established LSTM models to achieve the automatic detection of epilepsy	A mean sensitivity of 93.61% and a mean specificity of 91.85% were achieved on a long-term scalp EEG database in this article
Koya et al. [184]	EEG	Normal (*n* = 10)	LSTM	Emotion	Adopted LSTM to recognize and classify different emotions	In this article, the LSTM + CNN model outperformed traditional or deep learning models, achieving an accuracy of 64%
Lee et al. [185]	EEG	Normal (*n* = 10)	LSTM	Sleep stages	Detected drowsiness indicators using the LSTM method	The LSTM-CNN model in this article demonstrated an average accuracy of 85.6% and a kappa index of 0.77 for the three-class classification problem

*AD* Alzheimer’s disease, *ASD* autism spectrum disorders, *BDLDA* block diagonal LDA linear discriminant analysis, *CHB-MIT* Children’s Hospital Boston (CHB) and the Massachusetts Institute of Technology, *CNN* convolutional neural network, *CSP* common spatial patterns, *EEG* electroencephalography, *iEEG* intracranial electroencephalography, *LDA* linear discriminant analysis, *LSTM* long short-term memory, *PD* Parkinson’s disease, *PDD* Parkinson’s disease-related dementia, *RNN* recurrent neural network, *SVM* support vector machine, *SZ* schizophrenia, *BCI* brain-computer interface, *WC* wavelet coherence, *TD* typically developing, *EDF* European Data Format

#### CSP

The CSP algorithm uses a linear transformation to maximize the variance ratio of two signals after mapping, which is a common spatial-filtering algorithm used for multi-channel EEG analysis [186].

${X}_{1},{X}_{2}$ refer to the signal data of $\left(n,{T}_{1}\right),(n,{T}_{2})$ size, where $n$ is the number of channels and ${T}_{1},{T}_{2}$ are the length of the respective signal:

45 $$w={argmax}_{w}\frac{{\Vert w{X}_{1}\Vert }^{2}}{{\Vert w{X}_{2}\Vert }^{2}}$$

$w$ denotes the projection matrix, which can be solved using matrix diagonalization.

In contrast to other spatial feature extraction methods, the CSP method is simple and efficient, but it is only suitable for processing two categories of signal data.

#### Linear discriminant analysis (LDA)

LDA, a classical linear method, is mainly used to find features that characterize or separate two classes and is also applicable for the dimensionality reduction of data [186]. Regarding projection, the projected data have high cohesion and low coupling characteristics.

46 $$(J\left(w\right)=\frac{{\left({M}_{1}-{M}_{2}\right)}^{2}}{{S}_{1}+{S}_{2}})$$

47 $$\widehat{w}={argmax}_{w} J\left(w\right)$$

${S}_{1},{S}_{2}$ are intra-class scatters, ${\left({M}_{1}-{M}_{2}\right)}^{2}$ refers to the inter-class scatter, and $\widehat{w}$ represents the mapping matrix. Because LDA assumes that the data obey the Gaussian distribution, it does not perform satisfactorily in processing data with non-Gaussian distributions.

#### SVM

SVM is a class of generalized linear classifiers for the classification of binary data in a supervised learning manner [176]. SVM constructs a hyperplane in high-dimensional space to distinguish between two classes of data. Assuming that the dataset is $[\left({x}_{1},{y}_{1}\right),\left({x}_{2},{y}_{2}\right),\dots \left({x}_{n},{y}_{n})\right]$, wherein ${y}_{i}\in [-1, 1]$, the hyperplane is defined as:

48 $${w}^{T}x-b=0$$

The plane separating the two classes of data is as follows:

49 $${w}^{T}{x}_{i}+{w}_{0}=1{,w}^{T}{x}_{i}+{w}_{0}=-1$$

Here, ${w}^{T}$ represents the normal vector and $b$ denotes the offset, so the data interval is $2/\Vert w\Vert$. This method maximizes $2/\Vert w\Vert$ while ensuring that all data satisfy the conditions. Methods such as Lagrangian duals can be used to solve such constrained optimization problems, computing the hyperplane of the separated data. SVM performs poorly in resolving multi-classification problems.

#### CNN

The unique convolutional layer of CNN can effectively extract EEG signals and structural information in the spatio-temporal frequency domains [187–189].

For feature extraction, the dot product is completed using the input data with the filterbank region-by-region, and each kernel is scanned using step length, sharing equal weight. The resulting output is a set of K-dimensional feature maps.

50 $${Z}_{j}^{l}=\sigma \left(\sum {Z}_{j}^{l-1}*{W}_{ j,i}^{l}+{B}_{j}^{l}\right)$$

Here, ${B}_{j}^{l}$ signifies the $j$th deviation in the layer $l$, ${W}_{ j,i}^{l}$ refers to the weight matrix connecting with the feature map in the neighboring layer (${Z}_{j}^{l},{Z}_{j}^{l-1}$), $*$ represents the convolution operator, and $\sigma (\cdot )$ denotes the nonlinear activation function.

The extracted feature maps are recognized by a classifier, which often uses the cross-entropy loss function:

51 $$L\left({y}_{i},{\widehat{y}}_{i}\right)=\frac{1}{N}\sum_{\dot{i}=1}^{N}(-{y}_{i}log{\widehat{y}}_{i}-\left(1-{y}_{i}\right)\mathrm{log}\left(1-{\widehat{y}}_{i}\right))$$

Here, ${y}_{i}$ represents the sample’s true value and ${\widehat{y}}_{i}$ signifies the model-predicted value. CNN requires less preprocessing than other algorithms but also has the risk of overfitting.

#### RNN

The RNN model performs well for temporal signals, wherein connections between nodes generate directed or undirected graphs along the time series, effectively extracting feature information in the time dimension [190]. However, gradient explosion and gradient vanishing problems are present due to RNN’s structure of backpropagation through time. Later, LSTM was developed, which has broader applications than RNN.

#### LSTM

LSTM has improved on the problems of the RNN network [191] and selectively transmits data utilizing forgetting gates, input gates, and output gates.

The forgetting gate determines which information to remove from the state of the unit:

52 $${f}_{t}=\sigma \left({W}_{f}\left[{h}_{t-1},{x}_{t}\right]+{b}_{f}\right)$$

The input gate determines which values will be updated:

53 $${i}_{t}= \sigma \left({W}_{i}\left[{h}_{t-1},{x}_{t}\right]+{b}_{i}\right)$$

54 $${\widetilde{C}}_{t}=\mathit{tan}h\left({W}_{c}\left[{h}_{t-1},{x}_{t}\right]+{b}_{c}\right)$$

Then, the unit value state is updated based on the equations above:

55 $${C}_{t}={\mathrm{f}}_{t}*{C}_{t-1}+{i}_{t}*{\widetilde{C}}_{t}$$

Finally, the output gate determines which parts of the unit state will be the final output:

56 $${o}_{t}= \sigma \left({W}_{o}\left[{h}_{t-1},{x}_{t}\right]+{b}_{o}\right)$$

57 $${h}_{t}={o}_{t}*\mathrm{tanh}\left({C}_{t}\right)$$

wherein $\sigma$ signifies the sigmoid activation function that compresses numbers to the range 0, 1, $tanh$ denotes the hyperbolic tangent activation function that compresses numbers to the range − 1, 1, ${W}_{f}$, ${W}_{i}$, ${W}_{c}$, and ${W}_{o}$ are the weight matrixes, ${x}_{t}$ represents the input vector, ${h}_{t-1}$ represents past hidden states, and ${b}_{f}$, ${b}_{i}$, ${b}_{c}$, and ${b}_{o}$ are deviation vectors. LSTM has a slow training speed due to its performing and processing difficulties [192].

#### Joint application of EEG analysis and machine learning methods

The joint application of EEG analysis and machine learning methods has been an active area of research in neuroscience and disease diagnosis. EEG is a non-invasive method of measuring the electrical activity of the brain, and machine learning algorithms can be used to extract information from EEG signals to help diagnose various disorders and identify different brain states [193]. Machine learning algorithms have been developed to extract features from EEG signals, such as frequency bands, time–frequency representations, and connectivity measures [193]. These features can then be used to train machine learning models to classify different brain states or diagnose various disorders [193]. Machine learning algorithms have been developed to detect seizures in EEG signals with high accuracy [194] and classify EEG signals from patients with Alzheimer’s disease and healthy controls [195].

The joint application of EEG analysis and machine learning methods has several advantages in neuroscience and disease diagnosis. It allows for the identification of patterns in EEG signals that are difficult to detect using traditional methods. Machine learning algorithms can be used to extract features from EEG signals that are not easily visible to the human eye, such as subtle changes in frequency or amplitude [193]. These features can then be used to train machine learning models to classify different brain states or diagnose various disorders. Another advantage of this combination is that it can help reduce the subjectivity of EEG analysis. Traditional EEG analysis methods rely on visual inspection of the EEG signal by a trained expert, which can be time-consuming and subjective [193]. Machine learning algorithms can be used to automate the process of EEG analysis, reducing the time and subjectivity involved in the analysis [194].

Moreover, different models may be better suited to different aspects of the data. One model may be better at detecting certain types of patterns in the data, while another model may be better at classifying the data into different categories. By combining the strengths of different models, it is possible to create a more accurate and robust analysis [193] to reduce the risk of overfitting, and create a more generalizable analysis [196].

In summary, the joint application of EEG analysis and machine learning methods has great potential for the diagnosis of various disorders and the identification of different brain states. It has several advantages, including the ability to identify patterns in EEG signals that are difficult to detect using traditional methods, and the ability to reduce the subjectivity of EEG analysis.

## Discussion

**Fig. 4:**
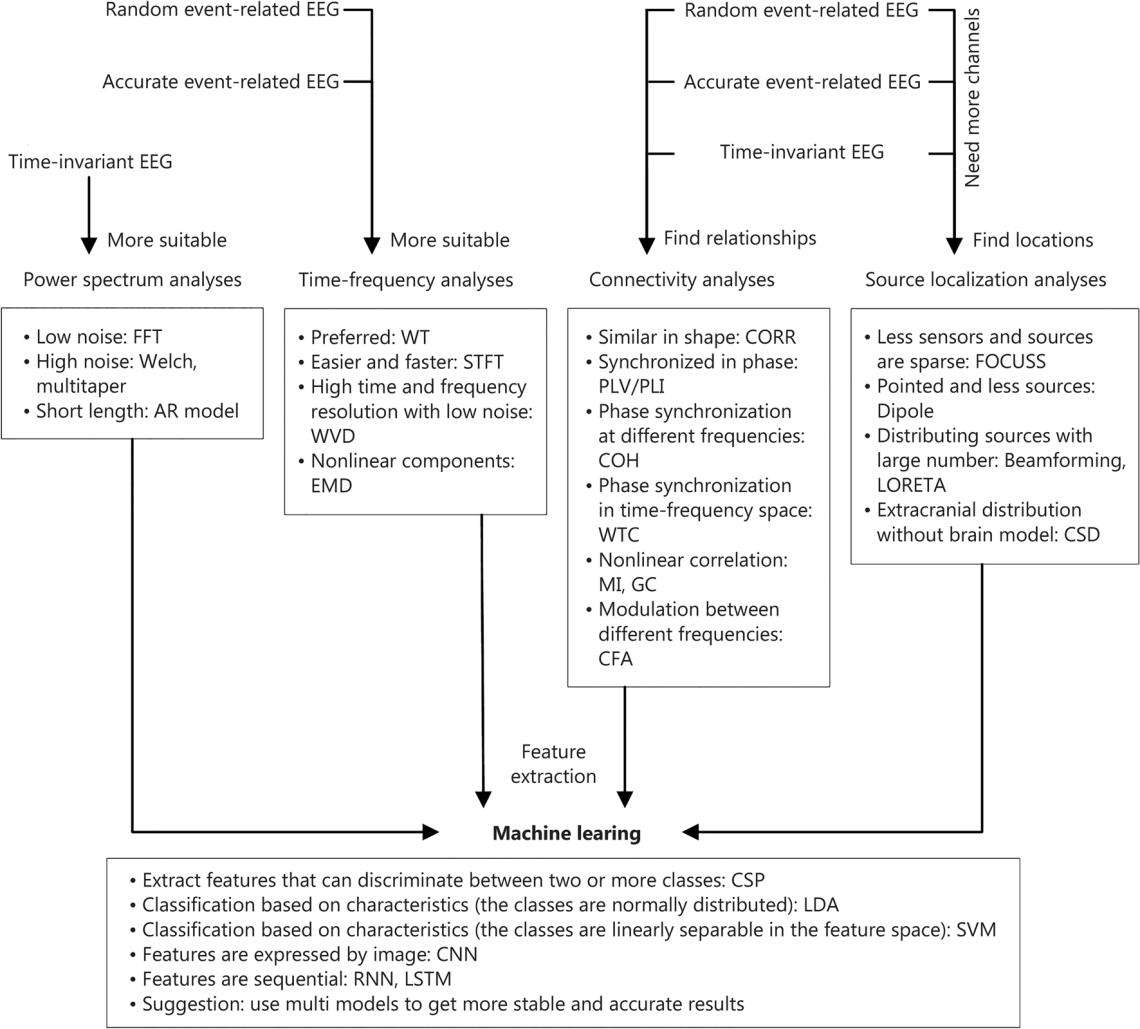
Summary of method selection for different data characteristics and application requirements. AR autoregressive, CFA ross-frequency analysis, CNN convolutional neural network, COH coherence, CORR correlation, CSD current source density, CSP common spatial patterns, EEG electroencephalography, EMD empirical mode decomposition, FFT fast Fourier transform, FOCUSS focal underdetermined system solution, GC granger causality, LDA linear discriminant analysis, LORETA low-resolution electromagnetic tomography, LSTM long short-term memory, MI mutual information, PLV/PLI phase lag value/index, RNN recurrent neural network, STFT short-time Fourier transform, SVM support vector machine, WT wavelet transform, WTC wavelet coherence, WVD Wigner-Ville distribution

This article reviewed several commonly used EEG/iEEG analysis methods in neuroscience and introduced the applied principles based on the data generation characteristics. Due to different EEG data generation approaches, there are fundamental differences in data time, events, and variability. Therefore, the methods for data analysis should be selected based on these characteristics to ensure theoretical accuracy. Figure 4 presents a summary diagram of method selection. The required method can be selected based on the characteristics of the EEG signal and application requirements. The various methods are discussed below.

The power spectrum analysis method is used to reflect the energy changes in various brain regions. FFT has a high-frequency resolution and accuracy but is easily affected by noise and requires a large amount of data [197]. Therefore, FFT is more suitable for analyzing time-invariant EEG signals of good quality [44]. Welch and multitaper can suppress noise in EEG signals using the window averaging method, but the frequency resolution is decreased and the requirement for data length is increased [198]. Hence, for time-invariant EEG, if the signals contain slight burst noise, Welch or multitaper is a good choice [45]. Additionally, for accurate event-related EEG, EEG can be segmented into epochs based on the onset time of events. Windowed superposition analysis of EEG in the same state, such as baseline EEG before stimulation, can also yield good power spectrum results. If the signal can be directly spliced, signal jumps easily occur at the splicing site, which can cause severe Gibs. Therefore, it is not recommended to use signal splicing before FFT calculation or Welch power spectrum analysis. The AR model can calculate the power spectrum after signal prediction and modeling, which is very suitable for analyzing short-term signal power spectra [58], such as the power spectrum of a small segment of rapidly changing EEG signals in event-related EEG. However, it should be noted that the AR model method is based on the model, so the selected model may not fit the signal well. Selection of the wrong model will result in large deviations [197]. The application of AR models requires a more accurate evaluation of the signals [199]. Additionally, for signals with rapid changes in signal amplitude, commonly used models cannot fit effectively, so AR models cannot effectively analyze the power spectrum. In summary, if the data obtained are of good quality, such as high-quality sleep data, FFT is recommended to obtain more accurate results. If the data contain random noise, such as in the long-term monitoring of epilepsy, Welch or multitaper is recommended. If the data are short, such as EEG after physical instant stimulation, the AR model is recommended for fitting. Power spectrum analysis is the basis of EEG signal analysis and one of the most important analysis methods. With upgrades in EEG acquisition equipment, the signal-to-noise ratio of the obtained EEG signal is also increasing. At this time, the transient power spectrum will play an important role, and related research will enhance the development of brain-computer interfaces (BCIs) and machine learning fields.

Time–frequency analysis is a powerful tool for analyzing event-related EEG signals because it can describe the changes in time-varying EEG from two dimensions based on the changes in time and frequency. Among the various time–frequency analysis methods, the continuous WT is the most commonly used method due to its good performance in balancing time and frequency. However, its high computational and spatial complexity makes it unsuitable for long-term data analysis [200]. Correspondingly, DWT shows a good performance in signal decomposition but a poor visualization effect and unsatisfactory frequency resolution [201]. For time-invariant EEG, although the changes in EEG are insignificant, the energy of diverse frequency bands will certainly change in response to long-term changes. STFT can present this response well, having good frequency resolution [3] and low time resolution [69]. If WT is used, although it is more informative, the resultant massive data redundancy is not conducive to observing the main features. WVD has a very high time–frequency resolution for short-term signals but is highly susceptible to noise. Therefore, WVD is more suitable for analyzing short-term signals with less noise [70]. EMD is very suitable for analyzing signals with many abrupt amplitudes [72] and will not be affected by Gibs, but WVD has poor frequency localization of low-noise signals [201]. With advances in computer performance, time–frequency analysis methods have gradually replaced power spectrum methods as the first choice for observing spectrum changes. The integration of time–frequency graphs and image-based deep learning has also produced many high-quality applications. However, it should be noted that time–frequency analysis is still not detailed enough for the calculation of instantaneous changes. For example, it is difficult to use time–frequency diagram analysis for the EEG signal during epileptic discharge. In this case, it should be combined with the time domain method to improve the description accuracy.

Connectivity analysis is an important part of neural signal analysis. The selection of relevant analysis methods is crucial to the main features that need to be analyzed. This article introduced several classical correlation analysis methods from the perspectives of time, frequency, and nonlinearity. However, many other methods have not been introduced, such as event statistics, CFC coupling, and amplitude frequency coupling. These methods are similar to the ones in this article and can be selected based on the data characteristics. Several correlation analysis methods applicable to long-term signals can be used to observe the correlations between different brain regions in time-invariant EEG due to insignificant changes in the characteristics of time-invariant EEG. CORR focuses more on the time-scale similarity of signals, while COH focuses more on their frequency-scale similarity [107]. The PLV/PLI method refers to phase-based statistics, with PLV being stricter. Such a method can be easily converted into correlation statistics of cross-frequency or other events [202]. These three methods can be used to measure the correlation between channels in terms of signal similarity and signal phase synchronization [112]. MI and GC can analyze the driving force between signals from the perspective of information transmission [109]. These two methods can be used to study the relationship between signals. MI observes the relationship from the perspective of information transmission, while GC infers the relationship from the perspective of regression. Both methods are nonlinear but differ in their approach. MI uses information entropy, while GC uses regression. As a result, the characteristics of the observations differ between the two methods [111]. However, WTC requires clear events to produce more accurate results, making it suitable for accurate event-related EEG but not for time-invariant EEG. All the methods mentioned above are also suitable for accurate event-related EEG. However, it is important to note that the relevant changes presented by event-related EEG during the event are accurate and real. Therefore, correlation analysis of short-term signals after the event is recommended to obtain related results with a higher signal-to-noise ratio. For random event-related EEG, due to the inaccuracy of its events, WTC methods are also unable to provide accurate results. However, due to the time-varying characteristics, other correlation analysis methods are required to segment and classify the signals according to the main analysis features to improve the signal-to-noise ratio of the results [203]. Connectivity analysis methods have emerged as a powerful tool for studying brain networks, which are important components of brain cognition. Moreover, these methods can be combined with neural networks to develop new bionic operations. Different connection patterns can represent different ways of connecting neurons. The existing neural networks usually use direct signal connections, but other connection methods can be used to produce more intelligent network interfaces. Spiking neural networks are a representative example of this type of research.

Source localization analysis can convert multi-channel EEG from the scalp to deeper brain regions, which can more clearly localize the position of signal generation. A personalized brain model is generated through structured MRI, following which intracranial nerve activity is inferred from extracranial nerve activity signals to estimate the discharge location of the personalized brain. This process can locate key intracranial locations without surgery and has been widely used in the fields of epilepsy focus location and functional area location. Existing source localization methods have different implementation principles. The minimum norm estimation can partly localize the source into the intracranial region but has low accuracy in deep source localization and a high requirement for signal quality due to the use of MEG [139]. The dipole and beamforming methods can effectively localize the source into the intracranial region and have good localization accuracy, but they require more accurate parameters and an accurate head model [142, 163]. Beamforming balances speed and accuracy and is a rapidly developing method [204]. The FOCUSS method has good resolution but low computational efficiency [140]. LORETA is currently the most frequently used source localization method [205]. The spatial resolution of this method is not high, but its temporal resolution is good; thus, it has received considerable attention in EEG analysis [139]. CSD does not require a brain model and its calculation process is simple and fast, but it cannot localize the source in the intracranial cavity [144]. Signal quality plays a crucial role in the accuracy of multi-channel brain source localization methods. Therefore, the source localization method best suits short-term EEG localization analysis in event-related EEG. By leveraging the high signal-to-noise ratio features at the onset time of events, more accurate localization results can be obtained. For the source localization analysis of time-invariant EEG, the discharge characteristics of the EEG should be converted before localization, or the superposition method should be used to increase the signal-to-noise ratio. For random event-related EEG, it is advisable to choose EEG signals with a high signal-to-noise ratio for localization as far as possible. Source location methods have significant restrictions, including signal quality, number of channels, and brain model accuracy, which can affect positioning accuracy. However, targeted selection can improve the accuracy of these methods. With the continuous development of computing power and artificial intelligence technology, the quality requirements of source localization methods will gradually decrease, allowing them to be widely used in clinical practice.

Machine learning methods are rapidly developing methods that learn features, which can be the original EEG signals or converted features of EEG [148], such as power and connectivity [149]. Hence, although machine learning has a wide range of adaptability due to its self-learning nature, the generation and selection of features remain points of discussion. For time-invariant EEG, the features of EEG can be the average of long-term features, such as the power spectrum and connectivity of each-channel EEG. For accurate event-related EEG, event-related features can be learned. For random event-related EEG, machine learning methods with clustering properties are more suitable for semi-supervised or unsupervised feature learning [150]. Deep learning has been successfully applied in multiple EEG signal tasks, such as motor imagery, epilepsy detection, severe depression detection, sleep stage scoring, and event-related potential tasks [206]. There are differences between the data for different tasks, such as signal window length and channel count. Given these differences, selecting the suitable type of deep learning network can achieve better classification performance. Using CNN to classify spectrograms can also produce good results [207], but CNN models are suitable for data classification without time information, while LSTM models are suitable for regression analysis with time information [190]. However, compared with traditional single models such as CNN and LSTM, a mixed model is recommended [208]. If a model fails to achieve the expected results, researchers can opt for a fusion of multiple models to improve accuracy. For skilled machine learning researchers, this is a simple and fast way to build applications. The mixed model is expected to perform well in classification accuracy and transfer learning. Nevertheless, deep learning models also have shortcomings. For example, due to the small size of disease datasets, the model is prone to overfitting. Therefore, methods such as introducing regularization terms into the model should be considered to minimize the impact of overfitting [209]. Moreover, the introduction of model interpretability can aid in understanding the feature selection for model classification. There may be causal relationships within the EEG features, and the introduction of causal algorithms can be considered to further optimize the models [210]. At present, most deep learning models are designed based on images and cannot adapt well to EEG signal data. The transformer, as a new type of neural network model, is being used in the diagnosis and treatment of brain diseases, but its application in EEG needs to be studied further [211]. Therefore, it is necessary to consider encoding and decoding the data based on EEG signal data features and developing new model structures. Machine learning methods are currently the fastest-growing neural signal processing methods, and many researchers have proposed new processing ideas in EEG analysis. With the rapid development of brain-like intelligence, a large-scale model may emerge to perform bionic simulations of human brain functions. This kind of research will help develop the field of artificial intelligence to a higher level.

This article discussed some commonly used EEG analysis methods. However, in practice, a combination of methods is frequently used. For instance, BCI, a frontier field of neuroscience and neurological diseases, requires the complex processing of brain electrical signals using multiple methods. A neural device known as a BCI translates the neural activity of an individual into external responses or directives. These interfaces find applications in restoring functionality for conditions such as epilepsy, stroke, spinal cord injuries, ALS, cerebral palsy, narcolepsy, Parkinson’s disease, and neuromuscular disorders [212]. In the realm of mental health, BCIs are under investigation as potential treatments for conditions like depression, anxiety, obsessive–compulsive disorder, and other neuropsychiatric disorders [213]. BCIs are versatile in acquiring a diverse array of signals, each associated with different objects. Despite this variability, the decoding of brain signals generally follows a five-stage process: signal acquisition, preprocessing, feature extraction, classification, and control interface. These stages involve the integration of various methodologies. Ongoing research in BCI analysis methods aims to enhance accuracy and reliability. Notably, the application of deep learning algorithms for EEG data analysis is a promising avenue. Another focus is on leveraging explainable artificial intelligence techniques to gain insights into BCI analysis outcomes. Like BCI, numerous studies necessitate the integration of traditional and innovative technologies to continually enhance the efficacy of EEG analysis methods and establish a foundation for further research.

## Conclusions

EEG/iEEG is commonly applied in functional neuroimaging and is one of the leading tools in neuroscience. Clinical medicine, BCIs, and psychological research all require EEG/iEEG analysis. In recent decades, a variety of analysis methods have emerged for researchers to choose from, and interest in such techniques is high. However, the abundance of analysis methods has led researchers to question their applicability.

This review categorizes representative research methods based on the characteristics of EEG/iEEG signals. The methods are classified into power spectrum analysis, time–frequency analysis, connectivity analysis, source localization analysis, and machine learning. Other methods with wide application scenarios, such as nonlinear analysis, predictive analysis, and graph theory analysis, are not introduced in this review. These methods are considered to have certain similarities to or be the extension of the classical methods in this review from the perspective of analysis purpose. The methods introduced in this review are only a subset of common methods, and users need to make choices based on the characteristics of the data and methods.

## Data Availability

Not applicable.

## Abbreviations

AR: Autoregressive
BCI: Brain-computer interface
CFA: Cross-frequency analysis
CFC: Cross-frequency coupling
CFD: Cross-frequency directionality
CNN: Convolutional neural network
COH: Coherence
CORR: Correlation
CSD: Current source density
CSP: Common spatial pattern
EEG: Electroencephalography
EMD: Empirical mode decomposition
FFT: Fast Fourier transform
FOCUSS: Focal underdetermined system solution
GC: Granger causality
iEEG: Intracranial electroencephalography
IMF: Intrinsic mode function
LDA: Linear discriminant analysis
LORETA: Low-resolution electromagnetic tomography
LSTM: Long short-term memory
MEG: Magnetoencephalography
MI: Mutual information
MRI: Magnetic resonance imaging
PLV/PLI: Phase lag value/index
RNN: Recurrent neural network
sLORETA: Standardized low-resolution electromagnetic tomography
STFT: Short-time Fourier transform
SVM: Support vector machine
WT: Wavelet transform
WTC: Wavelet coherence
WVD: Wigner-Ville distribution
